# Sphingosine kinase-2 prevents macrophage cholesterol accumulation and atherosclerosis by stimulating autophagic lipid degradation

**DOI:** 10.1038/s41598-019-54877-6

**Published:** 2019-12-04

**Authors:** Kazuhiro Ishimaru, Kazuaki Yoshioka, Kuniyuki Kano, Makoto Kurano, Daisuke Saigusa, Junken Aoki, Yutaka Yatomi, Noriko Takuwa, Yasuo Okamoto, Richard L. Proia, Yoh Takuwa

**Affiliations:** 1 Department of Physiology, Kanazawa University School of Medicine, Kanazawa, 920-8640 Japan; 2 Department of Biochemistry, Graduate School of Pharmaceutical Science, Tohoku University, Sendai, 980-8578, Japan; 3 Department of Laboratory Medicine, Graduate School of Medicine, University of Tokyo, Tokyo, 113-0033 Japan; 4 Department of Integrative Genomics, Tohoku University Tohoku Medical Megabank Organization, Sendai, 980-8573 Japan; 5 Department of Health and Medical Sciences, Ishikawa Prefectural Nursing University, Kahoku, 929-1210 Japan; 6 Genetics of Development and Disease Branch, National Institute of Diabetes and Digestive and Kidney Diseases (NIDDK), NIH, Bethesda, MD 20892 USA

**Keywords:** Atherosclerosis, Diseases, Molecular medicine

## Abstract

Atherosclerosis is the major cause of ischemic coronary heart diseases and characterized by the infiltration of cholesterol-accumulating macrophages in the vascular wall. Although sphingolipids are implicated in atherosclerosis as both membrane components and lipid mediators, the precise role of sphingolipids in atherosclerosis remains elusive. Here, we found that genetic deficiency of sphingosine kinase-2 (SphK2) but not SphK1 aggravates the formation of atherosclerotic lesions in mice with ApoE deficiency. Bone marrow chimaera experiments show the involvement of SphK2 expressed in bone marrow-derived cells. In macrophages, deficiency of SphK2, a major SphK isoform in this cell type, results in increases in cellular sphingosine and ceramides. SphK2-deficient macrophages have increases in lipid droplet-containing autophagosomes and autolysosomes and defective lysosomal degradation of lipid droplets via autophagy with an impaired luminal acidic environment and proteolytic activity in the lysosomes. Transgenic overexpression of SphK1 in SphK2-deficient mice rescued aggravation of atherosclerosis and abnormalities of autophagosomes and lysosomes in macrophages with reductions of sphingosine, suggesting at least partial overlapping actions of two SphKs. Taken together, these results indicate that SphK2 is required for autophagosome- and lysosome-mediated catabolism of intracellular lipid droplets to impede the development of atherosclerosis; therefore, SphK2 may be a novel target for treating atherosclerosis.

## Introduction

Atherosclerosis is the major cause of ischemic heart diseases and characterized by the accumulation of macrophage- and smooth muscle-derived foam cells that have taken up oxidized low-density lipoprotein (OxLDL) in the subendothelial layer of the vasculatures^1^. Foam cells release proinflammatory cytokines to induce continued local inflammation and endothelial cell damages, leading to intimal thickening, narrowing of the vascular lumen and finally its occlusion with thrombus formation. Foam cells are also susceptible to apoptosis, which contributes to plaque instability. Therapies for risk factors of atherosclerosis such as dyslipidemia, diabetes and hypertension are well established and have proven effective to some extents^2^. However, therapeutic strategy, which directly targets one of the important players, macrophages, in pathogenesis of atherosclerosis, has been lacking because of our incomplete understanding of the atherogenic mechanisms.

In OxLDL-loaded macrophages, cytoplasmic lipid droplets (LDs) are isolated into the autophagosomes, delivered to lysosomes, and hydrolysed by lysosomal acid lipase, which leads to the release of free cholesterol. The cholesterol is then transported into the cell exterior by the ABC family transporters located on the plasma membrane. Autophagy is an essential process for LD breakdown in macrophages and serves a protective role in cholesterol accumulation in macrophages and consequently atherosclerosis^3–5^.

Sphingolipids may also be involved in atherosclerosis^6^. Sphingomyelin, which is the most abundant sphingolipid species in the cell membrane, has a high affinity to cholesterol and accumulates in advanced atheroma^7^. Sphingomyelin and other sphingolipids located in the plasma membrane and the cell organelles are converted to ceramides, sphingosine and sphingosine-1-phosphate (S1P) by the sequential actions of the sphingolipid-metabolizing enzymes. S1P is established as the blood-borne, pleiotropic lipid mediator that acts through binding to the S1P-specific, cell surface G protein-coupled receptors S1PR1-S1PR5^8,9^. In mouse models of atherosclerosis, genetic knockout (KO) studies showed that S1PR2 and S1PR3 facilitate atherosclerosis^10,11^, whereas pharmacological stimulation of S1PR1diminishes atherosclerosis^12,13^.

S1P is produced from sphingosine by the actions of sphingosine kinase-1 (SphK1) and sphingosine kinase-2 (SphK2), which are solely responsible for generation of S1P *in vivo*^14^. Neither *Sphk1*-KO (*Sphk1*^*−/−*^) nor *Sphk2*-KO (*Sphk2*^*−/−*^) mice display any phenotypic abnormality under basal conditions, whereas double KO (*Sphk1*^*−/−*^; *Sphk2*^*−/−*^) mice are embryonic lethal and have almost complete absence of S1P in the fetal tissues^15^, suggesting that sphingolipid metabolism via SphKs is indispensable for embryonic development. In addition to synthesizing the lipid mediator S1P, SphKs are implicated in endocytosis^16,17^; lysosomal functions including Ca^2+^ release and regulation of the transcription factor TFEB^18^; and autophagy^19^, for which the detailed mechanisms are not fully understood.

In a mouse model of atherosclerosis, pharmacological inhibition of SphKs by a non-selective SphK inhibitor reduced plasma S1P concentration and exhibited both atherogenic and anti-atherogenic properties^20^. As a consequence, the compound had no significant effect on atherosclerotic lesion formation. However, it is possible that SphK1 and SphK2 have distinct, isoform-specific roles in atherosclerosis. For example, a recent study^21^ showed a SphK2-specific role in the regulation of ABCA1-mediated cholesterol efflux. In this study, we analysed atherosclerosis in Western diet (WD)-fed *Sphk1*^*−/−*^ mice and *Sphk2*^*−/−*^ mice, both with an ApoE-deficient background and found that *Sphk2*^*−/−*^ mice, but not *Sphk1*^*−/−*^ mice, show aggravation of atherosclerosis because of defective autophagic breakdown of LDs in macrophages. These observations indicate that SphK2 is a novel key factor essential for autophagosome- and lysosome-mediated LD catabolism and may be a target in the development of new therapies for atherosclerosis.

## Results

### Genetic disruption of *Sphk2* aggravates atherosclerosis in mice

**Figure 1:**
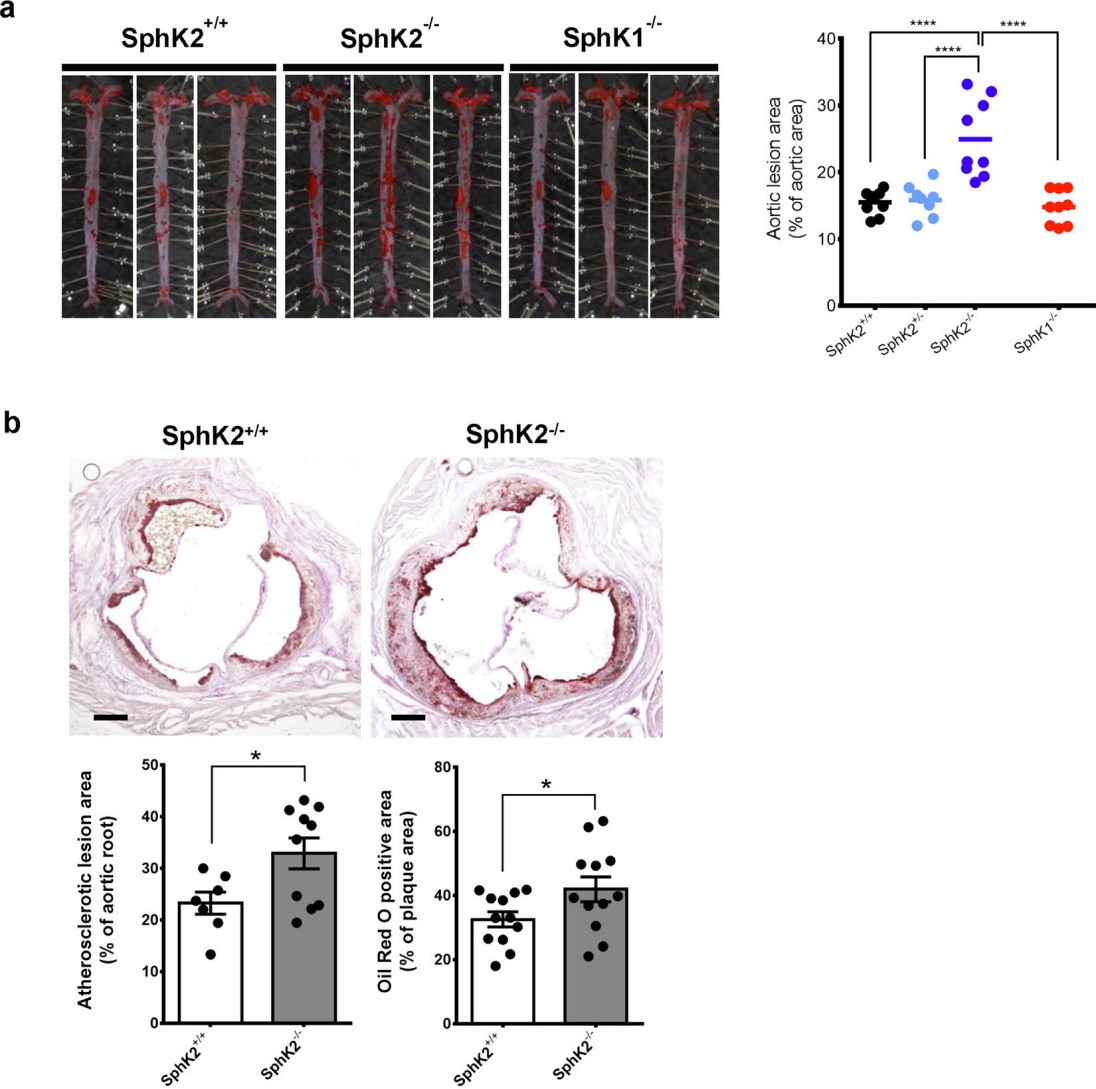
Aggravation of atherosclerosis in SphK2-, but not SphK1-, deficient mice. (**a**) En face aortic lesion areas stained with ORO from control *SphK1*^+/+^*; SphK2*^+/+^ (*SphK2*^+/+^), *SphK2*^+/−^, *SphK2*^−/−^, and *SphK1*^−/−^ male mice fed a WD for 12 weeks (n = 8–9 per group). (**b**) Atherosclerotic lesion size and lipid deposition in aortic roots from control *SphK2*^+/+^ mice and *SphK2*^−/−^ male mice, which were fed a WD for 12 weeks (n = 7–10 per group). Scale bars, 250 μm. *P < 0.05 and ****P < 0.0001.

To investigate roles of the two SphK isoforms in atherosclerosis, we fed four mice groups with different SphK genotypes, i.e. *Sphk1*^+/+^;* Sphk2*^+/+^ (hereafter abbreviated as *Sphk2*^+/+^), *Sphk1*^+/+^; *Sphk2*^+/−^ (*Sphk2*^+/−^), *Sphk1*^+/+^; *Sphk2*^−/−^ (*Sphk2*^−/−^), and *Sphk1*^−/−^;* Sphk2*^+/+^ (*Sphk1*^−/−^) in the Apoe^*−/−*^ background, on a WD for 12 weeks, followed by determinations of the aortic plaque lesion areas. The plaque lesions in the spread aortae, as evaluated with Oil Red O (ORO) staining, were increased by approximately 60% in *Sphk2*^*−/−*^ mice compared with control *Sphk2*^+/+^ mice, whereas those in *Sphk1*^−/−^ and *Sphk2*^+/−^ mice were similar to those in control mice (Fig. 1a). Total intimal lesion size and ORO-positive area in the cross-sections of the aortic sinus, a site frequently affected by atherosclerosis, were greater in *Sphk2*^*−/−*^ mice than control mice (Fig. 1b). The ORO-positive cross-sectional area of the abdominal aorta in *Sphk2*^*−/−*^ mice was also increased compared with control mice (Supplemental Fig. S1). Plasma concentrations of total cholesterol and triglyceride, plasma lipoprotein profiles, liver histology and cardiovascular parameters were all similar between *Sphk2*^*−/−*^ mice and control mice (Supplemental Figs. S2, S3a,b). Both groups of mice had similar body weights after 12 weeks of WD feeding, although the basal body weight of *Sphk2*^*−/−*^ mice at 8 weeks was slightly lower than that of control mice (Supplemental Fig. S3c). These results suggest that SphK2 has a protective role in atherosclerotic lesion formation in the aorta without affecting a plasma lipid profile.

### SphK2 deletion in bone marrow (BM)-derived cells aggravates atherosclerosis

**Figure 2:**
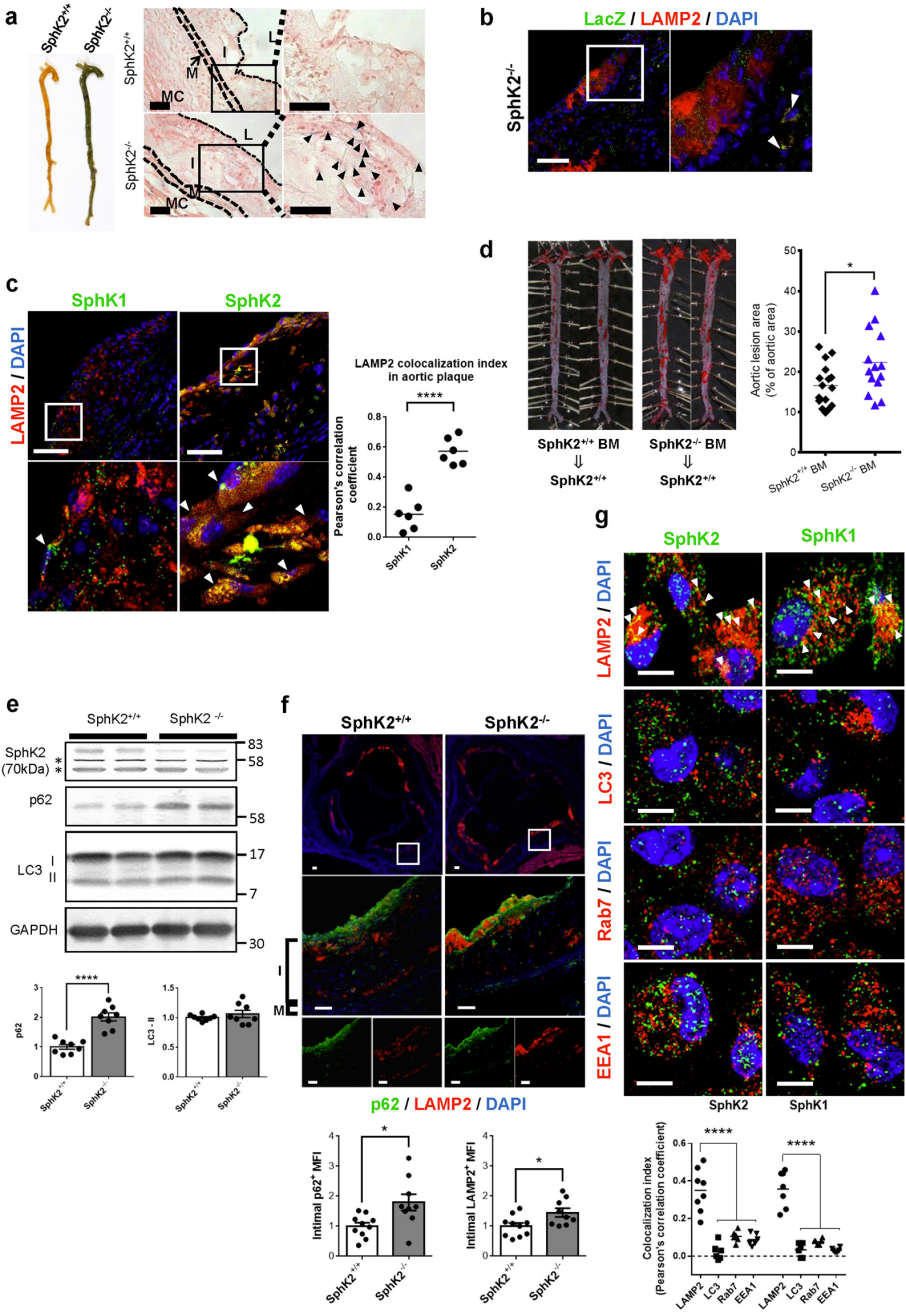
Expression of SphK2 in aortic plaques and aggravation of atherosclerosis in chimeric mice with SphK2 deletion in bone marrow-derived cells. (**a**) The detection of SphK2 expression by X-gal staining. Left, X-gal-stained whole aortae of *SphK2*^+/+^ and *SphK2*^−/−^ mice. Right, aortic sections. The arrowheads indicate X-gal-stained cells. L: lumen, I: intima, M: media, MC: myocardium. Scale bars, 50 μm. (**b**) LacZ expression in LAMP2-positive macrophages. Aortic root sections of *SphK2*^−/−^ mice after 12weeks of WD feeding were immunostained using anti-LacZ and anti-LAMP2 (macrophage marker) antibodies. Scale bars, 50 μm.Scale bars, 50 μm. (**c**) SphK1 and SphK2 expression in LAMP2-positive macrophages. Aortic root sections of *SphK2*^−/−^ mice after 12 weeks of WD feeding were immunostained using either anti-SphK1or anti-SphK2 and anti-LAMP2 antibodies. Scale bars, 50 μm. (**d**) Aortic lesions stained with ORO in BM chimeric mice fed a WD for 12 weeks (n = 14–16 per group). The arrowhead indicates LAMP2. (**e**) Immunoblot analysis of LC3, p62 and LAMP2 proteins in lysates of aortae from WD-fed mice. Representative blots (top). *Non-specific bands. The levels of these proteins were normalized to those of GAPDH (n = 8 per group) (bottom). (**f**) Immunostaining of aortic root sections after 12weeks of WD feeding (n = 9–10 per group). Scale bars, 50 μm. MFI: Mean fluorescent intensity. (**g**) Subcellular distribution of SphK1 and SphK2 in peritoneal macrophages from control SphK2^+/+^ male mice. The arrowheads indicate the localization of SphK1 and SphK2 in LAMP2-positive lysosomes. Scale bars, 5 μm. *P < 0.05 and ****P < 0.0001.

We investigated SphK2 gene expression in various mouse tissues by performing 5-bromo-4-chloro-3-indolyl β-D-galactopyranoside (X-gal) staining of tissues from *Sphk2*^*−/−*^ mice that harbor the β-galactosidase (LacZ) gene at the SphK2 gene locus^15^. Macroscopically, *Sphk2*^*−/−*^ mouse aortae showed much more intense blue color in X-gal staining compared with *Sphk2*^+/+^ mouse aortae (Fig. 2a, left). Microscopically, X-gal-positive cells (arrowheads in Fig. 2a) were scattered in the intima of aortic plaques. The double immunostaining of aortic plaques using anti-monocyte/macrophage marker LAMP2/Mac3 and anti-LacZ antibodies showed LacZ immunoreactivity in LAMP2-positive macrophages (Fig. 2b). Moreover, anti-SphK1 and anti-SphK2 immunostaining of aortic plaques showed that SphK2 was stained as fine and coarse puncta and larger dots with the strong colocalization of SphK2 and the lysosomal membrane protein LAMP2, whereas SphK1 was stained in a coarse punctate pattern in LAMP2-positive cells with modest colocalization between SphK1 and LAMP2 (Fig. 2c). The intimal cells were positive for the macrophage marker F4/80 (Supplemental Fig. S4). These observations suggested that macrophages in the plaque expressed SphK2. Therefore, we studied a role of SphK2 in BM-derived cells by generating chimeric mice in which *Sphk2*^+/+^ host mice had received transplantation of BM cells from *Sphk2*^*−/−*^ or control donor mice (Fig. 2d). The ORO-stained atherosclerotic lesion area in the spread aortae was greater in mice that received *Sphk2*^*−/−*^ BM compared with mice that received *Sphk2*^+/+^ BM. These observations suggest that SphK2 in BM-derived cells has a protective role in atherosclerosis.

Recent studies^3–5^ reported a protective role of autophagy in limiting cholesterol accumulation in macrophages and attenuation of autophagy in plaque macrophages. Moreover, a homeostatic role of sphingosine kinases in autophagy and lysosomal functions in macrophages and other cell types was suggested^19,22^. Deficiency of SphK2 or both SphK1 and SphK2 in macrophages resulted in enhancement of autophagic vesicles, which might be due to abnormal lysosomes or enhanced autophagy^22^. Therefore, we studied possible impairment of autophagy in *Sphk2*^*−/−*^ mice. In tissue lysates from the aortae of WD-fed *Sphk2*^*−/−*^ mice, the expression of p62 (Sqstm1), a ubiquitin-binding scaffold protein which recruits ubiquitinated autophagosomal contents to the phagophore isolation membrane by binding to phagophore-bound LC3^3^, was increased compared with control mice (Fig. 2e), suggesting impaired progress of autophagic degradation processes in the aortae of *Sphk2*^*−/−*^ mice. Furthermore, double immunofluorescence staining of aortic sections showed enhanced accumulation of LAMP2-positive macrophages and an increase of p62 expression in LAMP2-positive plaque macrophages (yellow) in *Sphk2*^*−/−*^ mice compared with control mice (Fig. 2f). Previous studies^16–19^ showed that both SphK1 and SphK2 were localized in the cytosol and cell organelles, which include lysosomes and early and late endosomes. Immunostaining of peritoneal macrophages showed that both SphK1 and SphK2 were highly co-localized in LAMP2-positive lysosomes in macrophages (Fig. 2g, arrowheads). These observations together suggest that aggravation of atherosclerosis in *SphK2*^*−/−*^ mice may be accompanied by defective autophagy in plaque macrophages.

Sphingosine level in macrophages was elevated approximately 4-fold in *Sphk2*^*−/−*^ mice compared with control mice (Supplemental Fig. S5a). Those of dihydrosphingosine and ceramides (16:0, 18:0, 20:0, 22:0 and 24:0) were also significantly increased in *Sphk2*^*−/−*^ macrophages compared with *Sphk2*^+/+^ macrophages. The plasma S1P concentration in normal chow-fed *Sphk2*^*−/−*^ mice was higher than that in control mice (Supplemental Fig. S5b), which is in agreement with previous reports^23,24^. Feeding *Sphk2*^*−/−*^ mice with a WD resulted in a further increase in plasma S1P, which was also consistent with a previous report^25^. The gene expression of S1P receptors and sphingolipid-metabolizing enzymes except SphK2 was similar or slightly increased in *SphK2*^*−/−*^ macrophages compared with *SphK2*^+/+^ macrophages (Supplemental Fig. S5c).

### Lipid-loaded autophagosomes and autolysosomes are increased in *Sphk2*^*−/−*^ macrophages

**Figure 3:**
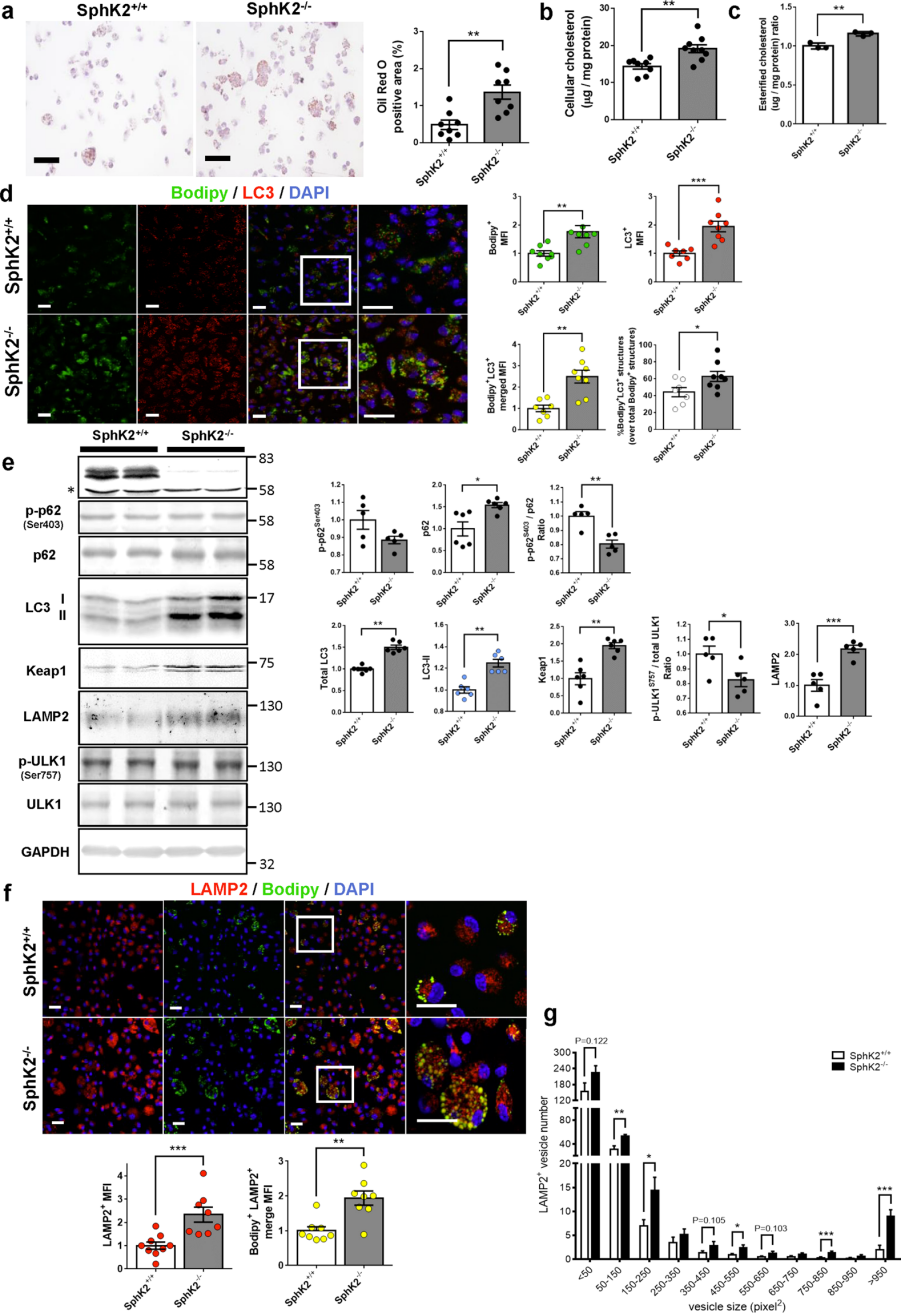
Increased cholesterol accumulation and impaired autophagy in SphK2^−/−^ macrophages. Peritoneal macrophages were freshly harvested from *SphK2*^+/+^ and *SphK2*^−/−^ male mice after 2 weeks of WD feeding. (**a**) Lipid deposition in ORO-stained *SphK2*^+/+^ and *SphK2*^−/−^ macrophages (n = 4 per group). Representative images (left). Scale bars, 50 μm. Quantified data of ORO-stained areas (n = 4 per group) (right). (**b**) The total cholesterol content of peritoneal macrophages from *SphK2*^+/+^ and *SphK2*^−/−^ male mice (n = 8–9 per group). (**c**) The esterified cholesterol content of peritoneal macrophage from *SphK2*^+/+^ and *SphK2*^−/−^ male mice (n = 3 per group). The data are expressed as a relative value to that of *SphK2*^+/+^ macrophages. (**d**) Lipid deposition in LC3-positive structures in macrophages from *SphK2*^+/+^ and *SphK2*^−/−^ male mice (n = 9–10 per group). Representative images (top). Scale bars, 20 μm. MFI of Bodipy- and anti-LC3 signals (bottom). Scale bars, 20 μm. (**e**) Immunoblot analysis of the autophagy-related protein expression in peritoneal macrophages from *SphK2*^+/+^ and *SphK2*^−/−^ male mice. Representative blots (left). *Non-specific band. The levels of these proteins were normalized to those of GAPDH (n = 5–6 per group) (right). (**f**) Lipid deposition in LAMP2-positive structures in macrophages from *SphK2*^+/+^ and *SphK2*^−/−^ male mice (n = 9–10 per group). Representative images (top). Scale bars, 20 μm. MFI of anti-LAMP2 signals, as well as Bodipy- and anti-LAMP2 double signals (bottom). (**g**) Histogram analysis of the sizes of LAMP2^+^ vesicles in macrophages. *P < 0.05, **P < 0.01 and ***P < 0.001.

We determined the lipid deposition in peritoneal macrophages freshly harvested from WD-fed *Sphk2*^*−/−*^ and *Sphk2*^+/+^ mice. Macrophages from *Sphk2*^*−/−*^ mice showed greater areas of ORO-positive LDs (Fig. 3a) and contained higher total cellular cholesterol (Fig. 3b) and esterified cholesterol (Fig. 3c), compared with macrophages from *Sphk2*^+/+^ mice. Treatment of macrophages with S1P treatment of *Sphk2*^+/+^ macrophages did not affect cholesterol content while S1P treatment of *Sphk2*^*−/−*^ macrophages only slightly increased cholesterol content (Supplemental Fig. S6).

Uptake of modified lipids by scavenger receptors and cholesterol efflux by ABC family transporters affect lipid deposition in macrophages. *Sphk2*^*−/−*^ macrophages have elevated basal cellular lipids compared with *Sphk2*^+/+^ macrophages, and *Sphk2*^+/+^ macrophages had 2.4-fold increase in cellular lipids after 4 h incubation with oxLDL whereas *Sphk2*^*−/−*^ macrophages had no increase in cellular lipid content (Supplemental Fig. S7a). The cholesterol efflux in the presence of 2% serum or HDL was not different between *Sphk2*^*−/−*^ and *Sphk2*^+/+^ macrophages (Supplemental Fig. S7b). OxLDL loading induced increases in the protein expression of cholesterol transporters ABCA1 and ABCG1. However, the extents of their expression were similar in *Sphk2*^*−/−*^ and *Sphk2*^+/+^ macrophages with oxLDL loading (Supplemental Fig. S7c).

We studied autophagic processes involved in LD catabolism in macrophages. LC3 in the cytosol (LC3-I) is recruited to the autophagosomes through its linkage to phosphatidylethanolamine in autophagosomal membranes, and the phosphatidylethanolamine-linked form of LC3 (LC3-II) shows a faster mobility on SDS-PAGE than LC3-I. Macrophages freshly harvested from WD-fed *Sphk2*^*−/−*^ mice had greater LC3-positive autophagosomes/autolysosomes and LD-containing (Bodipy 493/503-positive), LC3-positive autophagosomes/autolysosomes, compared with macrophages from *Sphk2*^+/+^ mice (Fig. 3d). Circulating foam cells of the monocyte lineage were also increased in atherogenic *Sphk2*^*−/−*^ mice (Supplemental Fig. S8). Autophagosome fusion with lysosomes and subsequent lipid digestion by lysosomal enzymes is the last step of autophagic lipid degradation^3–5^. Macrophages from WD-fed *Sphk2*^*−/−*^ mice showed increases in non-degraded total p62, its cytosolic sequester Keap1 and LC3-II, suggesting a constitutively impaired autophagic flux (Fig. 3e). Chloroquine treatment of macrophages increased the levels of LC3-II protein in Sphk2^+/+^ and Sphk2^*−/−*^ macrophages (Supplemental Fig. S9a). The difference before and after the CQ treatment (autophagic flux) seemed to be greater in SphK2^+/+^ macrophages. p62 is subjected to phosphorylation at Ser^403^, which activates the autophagosomal content-recruiting activity of p62^3^. The fractional phosphorylation of p62 (p-p62/total p62) in *Sphk2*^*−/−*^ macrophages was mildly decreased compared with *Sphk2*^+/+^ macrophages (Fig. 3e), suggesting that the process of LD recruitment into the autophagosomes might be slightly reduced in *Sphk2*^*−/−*^ macrophages compared with *Sphk2*^+/+^ macrophages. ULK1 is necessary for initiating the phagophore formation, the initial step of autophagosome formation, and phosphorylation at Ser^757^ of ULK1 by mTOR abolishes the phagophore formation-stimulating activity of ULK1^3^. The fractional phosphorylation of ULK1 (p-ULK1/total ULK1) in *Sphk2*^*−/−*^ macrophages was slightly decreased compared with *Sphk2*^+/+^ macrophages (Fig. 3e), suggesting that the phagophore formation might be rather slightly enhanced in *Sphk2*^*−/−*^ macrophages compared with *Sphk2*^+/+^ macrophages. Immunostaining showed increased LAMP2-positive structures in *Sphk2*^*−/−*^ macrophages compared with *Sphk2*^+/+^ macrophages (Fig. 3f). It is noteworthy that Bodipy- and LAMP2-double positive structures, lipid-containing autolysosomes/lysosomes, were more abundant in *Sphk2*^*−/−*^ macrophages than *Sphk2*^+/+^ macrophages. Histogram analysis of the size distribution revealed that the size of individual LAMP2-positive structures in *Sphk2*^*−/−*^ macrophages was greater than in *Sphk2*^+/+^ macrophages (Fig. 3g).

### Lysosomal functions and autophagic breakdown of LDs are impaired in *SphK2*^*−/−*^ macrophages

**Figure 4:**
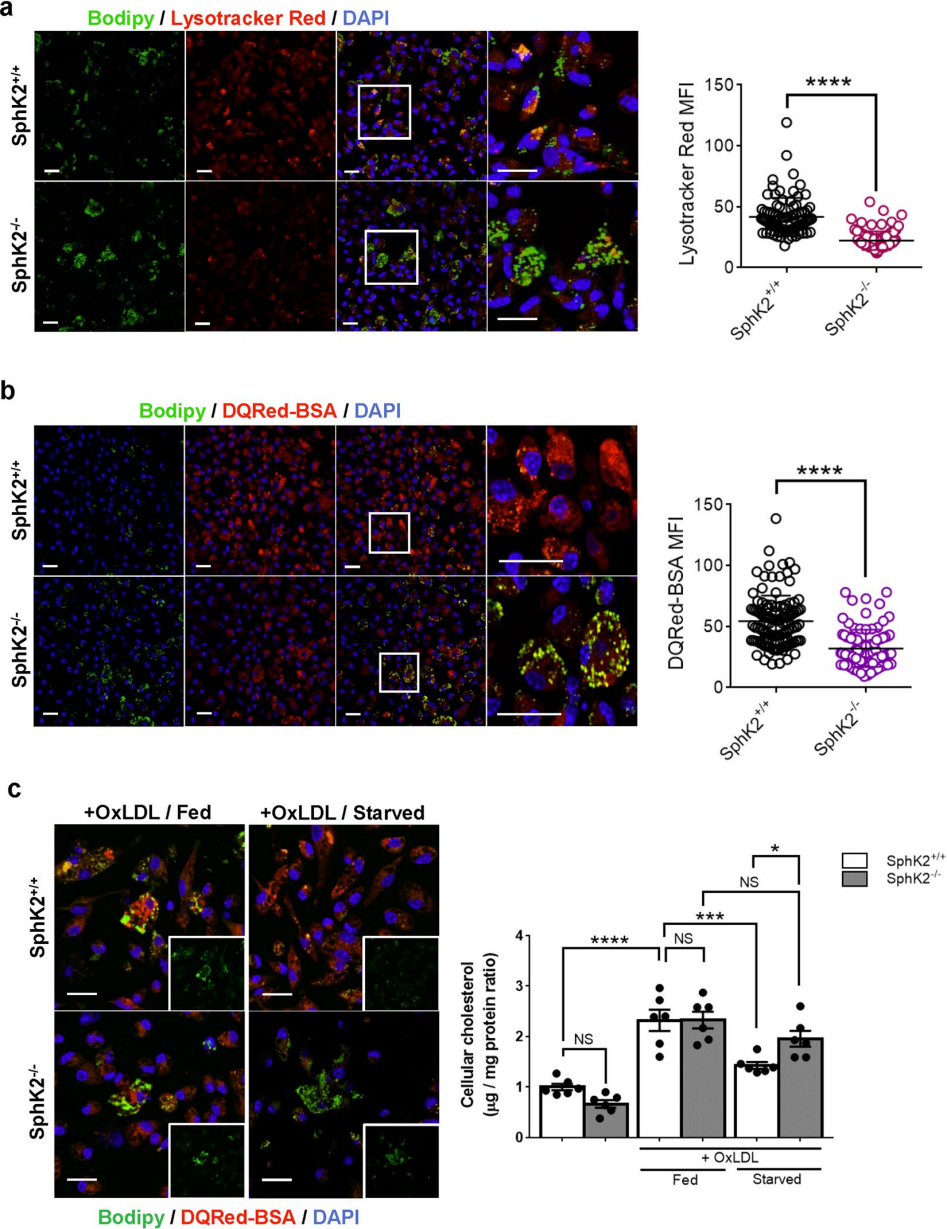
Impaired lysosomal activity in SphK2^−/−^ macrophages. Peritoneal macrophages were freshly harvested from *SphK2*^+/+^ and *SphK2*^−/−^ male mice after 2 weeks of WD feeding. **(a**) Staining of macrophages with the pH-sensitive LysoTracker Red and Bodipy. (**b**) Staining of macrophages with DQRed-BSA and Bodipy. (**a**,**b**) n = 5–6 mice per group. Total observed cell numbers were 88–117. Representative images (left). Scale bars, 20 μm. MFI of LysoTracker Red- and DQRed-BSA signals (right). (**c**) Effects of starvation on lipid deposition in macrophages. Peritoneal macrophages freshly harvested from non-WD fed mice were loaded with or without OxLDL for 4 h, followed by culture in normal growth medium (fed) or amino acid-depleted medium (starved) for 4 h (n = 6 per group). Representative images (left). Scale bars, 20 μm. Cellular cholesterol content (right). *P < 0.05, ***P < 0.001 and ****P < 0.0001. NS, not significant.

To monitor functional lysosomes in macrophages, we stained macrophages with LysoTracker dye, which is a pH-sensitive dye and labels acidic organelles including lysosomes, late endosomes and autolysosomes. Despite that LAMP2-positive structures were increased in *Sphk2*^*−/−*^ macrophages (Fig. 3f), LysoTracker fluorescence intensity in *SphK2*^*−/−*^ macrophages was decreased compared with *Sphk2*^+/+^ macrophages (Fig. 4a), which suggested that the luminal pH in the acidic organelles of *Sphk2*^*−/−*^ macrophages was not as low as that in *Sphk2*^+/+^ macrophages (Fig. 4a). Similarly, visualization of lysosomal proteolytic activities with DQRed-BSA showed decreased fluorescence in *Sphk2*^*−/−*^ macrophages compared with *Sphk2*^+/+^ cells (Fig. 4b), indicating a reduced lysosomal proteolytic activity in *Sphk2*^*−/−*^ macrophages. Furthermore, *Sphk2*^*−/−*^ macrophages had enhanced accumulation of Bodipy-stained LDs in DQRed-BSA-positive lysosomes than *Sphk2*^+/+^ macrophages. When autophagy was stimulated in OxLDL-loaded macrophages by amino acid starvation, *Sphk2*^+/+^ macrophages showed a reduction in Bodipy-stained LDs, whereas *Sphk2*^*−/−*^ macrophages did not (Fig. 4c left). Accordingly, starvation reduced cellular cholesterol content in *Sphk2*^+/+^ macrophages but not in *Sphk2*^*−/−*^ macrophages (Fig. 4c right). These data suggest that *Sphk2*^*−/−*^ macrophages have lysosomal dysfunctions and impaired autophagic LD degradation, which leads to accumulation of LDs in the autolysosomes.

To investigate the mechanisms underlying the altered autophagic LD catabolism in *Sphk2*^*−/−*^ macrophages, we analyzed the gene expression in macrophages freshly harvested from WD-fed mice. Interestingly, *Sphk2*^*−/−*^ macrophages showed 20–60% increases in the expression of lysosome-related genes, including the H^+^-ATPase Atp6vod2, the lysosomal acid lipase LIPA and the lysosomal glycosylated membrane protein Lamp2, as well as the autophagy-related genes LC3B and p62 and the lipid oxidase stress-activated transcription factor NRF2 (Supplemental Fig. S9b). Concomitantly, mRNA expression of the lysosome- and autophagy-regulating master transcription factor TFEB^26^ was elevated in *Sphk2*^*−/−*^ macrophages. These findings indicate that the expression of lysosome- and autophagosome-related genes was not decreased but upregulated in *Sphk2*^*−/−*^ macrophages. Despite that, *Sphk2*^*−/−*^ macrophages had dysfunctions at the lysosomes/autolysosomes and impaired LD degradation. Previous studies^26,27^ showed that the phosphorylation status regulates the activity of TFEB: the dephosphorylated form of TFEB is active and translocates into the nucleus to stimulate transcription of its target genes. The major phosphorylating kinase of TFEB is mTOR. Indeed, the mTOR inhibitor Torin abolished phosphorylation of TFEB in macrophages (Supplemental Fig. S9c). However, the activity of mTOR appeared to be rather increased in *Sphk2*^*−/−*^ macrophages compared with *Sphk2*^+/+^ macrophages, as evaluated by phosphorylation of the endogenous mTOR substrate 4E-BP1. Nevertheless, the ratio of dephosphorylated TFEB to total TFEB was not different between *Sphk2*^*−/−*^ and *Sphk2*^+/+^ macrophages (Supplemental Fig. S9c,d). Therefore, it is possible that SphK2 could be involved in the regulation of TFEB activity in a manner not directly dependent on the phosphorylation event of TFEB.

Impaired autophagy was also reported to be linked via inflammasomes^28^ to the enhanced production of inflammatory cytokines, especially interleukin (IL)-1β, which is implicated in atherogenesis^29^. Although the level of proIL-1β protein in *Sphk2*^*−/−*^ macrophage was increased compared with *Sphk2*^+/+^ macrophages, the levels of mature IL-1β protein within the cells and in the culture media were not different between *Sphk2*^*−/−*^ and *Sphk2*^+/+^ macrophages (Supplemental Fig. S9e). WD-feeding by itself did not alter the plasma IL-1β concentration in either *Sphk2*^*−/−*^ or *Sphk2*^+/+^ mice (Supplemental Fig. S9f). However, the challenge of mice with lipopolysaccharide (LPS) resulted in an approximately two-fold greater increase in plasma IL-1β in WD-fed *Sphk2*^*−/−*^ mice than in WD-fed *Sphk2*^+/+^ mice.

### Overexpression of SphK1 restores macrophage autophagic dysfunction and attenuates atherosclerosis in *Sphk2*^*−/−*^ mice

**Figure 5:**
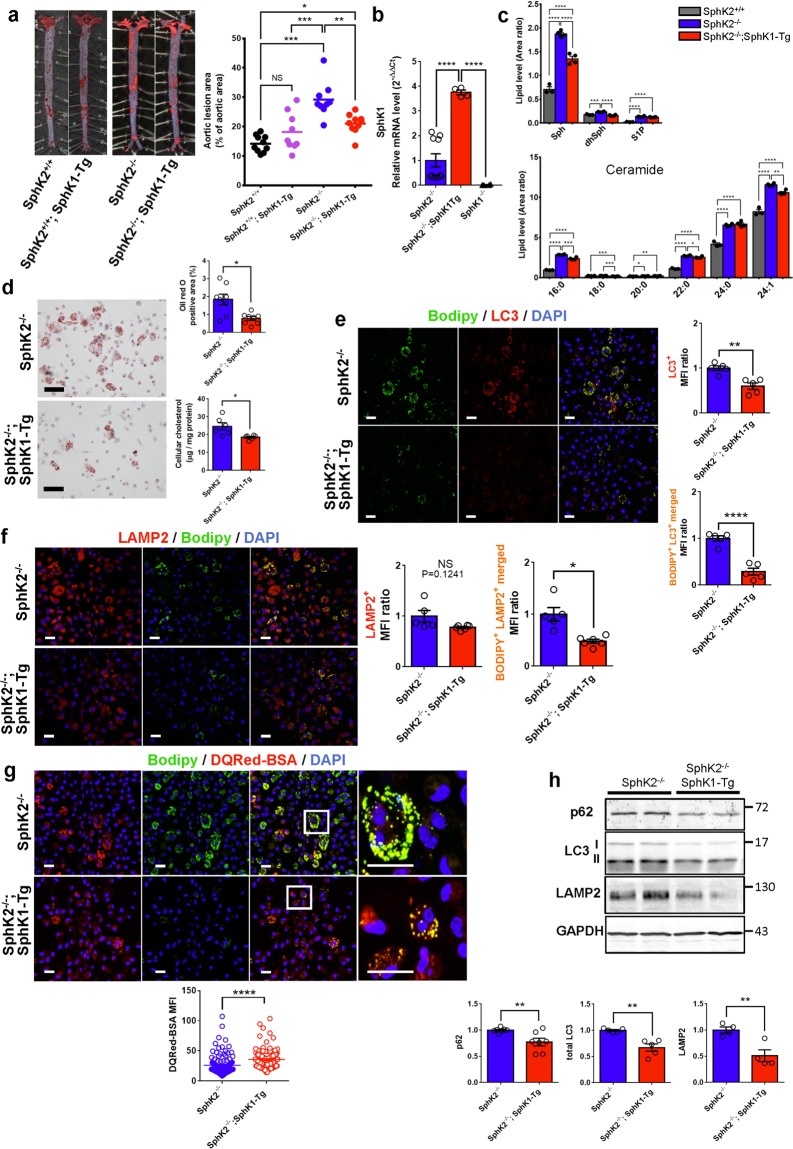
Effects of SphK1 overexpression on atherosclerosis, sphingolipid accumulation and autophagic dysfunctions in SphK2^−/−^ mice. (**a**) En face aortic lesion area stained with ORO from *SphK2*^+/+^, *SphK1-Tg*, *SphK2*^−/−^, and *SphK2*^−/−^*; SphK1-Tg* male mice fed a WD for 12 weeks (n = 9–10 per group). (**b**) SphK1 mRNA expression in macrophages from *SphK2*^−/−^, *SphK2*^−/−^*; SphK1-Tg*, and *SphK1*^−/−^ mice (n = 4–10 per group). (**c**) Cellular levels of sphingosine (Sph), dihydrosphingosine (dhSph), S1P and ceramides in macrophages from *SphK2*^+/+^, *SphK2*^−/−^, and *SphK2*^−/−^*; SphK1-Tg* mice (n = 3–4 per group). (**d**) Lipid deposition in macrophages from *SphK2*^−/−^ and *SphK2*^−/−^*; SphK1-Tg* male mice (n = 4 per group). Representative images (left). Scale bars, 50 μm. ORO-stained areas and cellular cholesterol content (right). (**e**) Lipid deposition in LC3-positive structures in macrophages from *SphK2*^−/−^ and *SphK2*^−/−^*; SphK1-Tg* male mice (n = 5–6 per group). Representative images (left). Scale bars, 20 μm. MFI of Bodipy- and LC3-positive signals (right). (**f**) Lipid deposition in LAMP2-positive structures in macrophages from *SphK2*^−/−^ and *SphK2*^−/−^*; SphK1-Tg* male mice (n = 5–6 per group). Representative images (left). Scale bars, 20 μm. MFI of anti-LAMP2 signals, as well as of Bodipy- and anti-LAMP2 double signals (right). (**g**) Staining of macrophages from *SphK2*^−/−^ and *SphK2*^−/−^; *SphK1-Tg* male mice with DQRed-BSA and Bodipy (n = 5–6 mice per group, total cell number = 119–128). Representative images (left). Scale bars, 20 μm. MFI of DQRed-BSA signals (right). (**h**) Immunoblot analysis of the autophagy-related protein expression in peritoneal macrophages. In (**b**–**h**), macrophages were freshly harvested from mice of the indicated genotypes, which were fed a WD for two weeks. *P < 0.05, **P < 0.01, ***P < 0.001 and ****P < 0.0001. NS, not significant.

There was a slight compensatory increase in SphK1 mRNA in *Sphk2*^*−/−*^ mouse macrophages (Supplemental Fig. S5c). We assessed whether SphK1 overexpression can rescue atherosclerosis in *Sphk2*^*−/−*^ mice. We generated *Sphk2*^*−/−*^ mice with transgenic overexpression of SphK1 (*Sphk2*^*−/−*^; *Sphk1-Tg*). Notably, compared with *Sphk2*^*−/−*^ mice, the compound mutant mice showed significantly reduced plaque areas (Fig. 5a). Transgenic SphK1 overexpression alone did not alter plaque areas. The mRNA level of SphK1 in macrophages from *Sphk2*^*−/−*^; *Sphk1-Tg* mice was 3.7-fold higher compared with that in *Sphk2*^*−/−*^ macrophages (Fig. 5b). The levels of sphingosine and dihydrosphingosine in macrophages from *Sphk2*^*−/−*^; *Sphk1-Tg* mice were reduced compared with *Sphk2*^*−/−*^ mice but was higher compared with *Sphk2*^+/+^ mice. The levels of ceramides including 16:0, 18:0, 22:0, 24:1 in macrophages from *Sphk2*^*−/−*^; *Sphk1-Tg* mice were also partially reduced compared with *Sphk2*^*−/−*^ mice. Macrophages freshly harvested from the compound mutant mice exhibited decreased accumulation of ORO-stained lipids and cellular cholesterol content compared with *Sphk2*^*−/−*^ macrophages (Fig. 5c). LC3-positive, LD-containing autophagosomes/autolysosomes were reduced in *Sphk2*^*−/−*^; *Sphk1-Tg* macrophages compared with *Sphk2*^*−/−*^ macrophages (Fig. 5d). The lysosome marker LAMP2-positive, LD-containing lysosomes/autolysosomes were also decreased in *Sphk2*^*−/−*^; *Sphk1-Tg* macrophages compared with *Sphk2*^*−/−*^ macrophages (Fig. 5e). Furthermore, the lysosomal proteolytic activity, as evaluated by DQRed-BSA fluorescence, was higher in *Sphk2*^*−/−*^; *Sphk1-Tg* macrophages compared with *Sphk2*^*−/−*^ macrophages (Fig. 5f). Consistent with these observations, the expression of LAMP2 and the autophagosomal and autolysosomal proteins p62 and LC3-II was reduced in macrophages from *Sphk2*^*−/−*^; *Sphk1-Tg* mice (Fig. 5g). Taken together, these data suggest that transgenic overexpression of SphK1 restores lysosomal and autolysosomal functions in macrophages to prevent accumulation of LD-containing autophagosomes/autolysosomes in *Sphk2*^*−/−*^ mice, leading to inhibition of atherosclerotic plaque formation.

## Discussion

Here, we demonstrate that lipid-loaded *Sphk2*^*−/−*^ but not *Sphk1*^*−/−*^ mice have larger atherosclerotic lesions than control mice. Mechanistically, *Sphk2*^*−/−*^ macrophages have increased lipid-containing autophagosomes/autolysosomes with the lysosomal dysfunctions, which include a reduced proteolytic activity and luminal acidification, suggesting impaired lysosomal degradation of the autophagic bodies. Moreover, the effects of genetic *Sphk* disruption on plaque formation are SphK isoform-specific, which is likely due to the predominant expression of the SphK2 isoform in macrophages because transgenic overexpression of SphK1 rescued the phenotypes caused by SphK2 deletion. Collectively, SphK2 plays a crucial role in the LD catabolism by autophagy and lysosomes in macrophages and has a protective role in atherogenesis.

In lipid-loaded mice, macrophages and smooth muscle in the vascular subendothelial layer uptake OxLDL and store OxLDL-derived cholesterol as esterified cholesterol in the intracellular LDs to become foam cells, which are a key component of atherosclerotic lesions. SphKs were previously shown to regulate hepatic lipid metabolism and adipogenesis^30,31^. However, SphK2 deficiency did not affect plasma lipoprotein profiles in this study. Notably, SphK2 deficiency resulted in increases in lipid-containing autophagosomes/autolysosomes in macrophages; nevertheless, cholesterol efflux was not altered by the SphK2 deficiency (Supplemental Fig. S7). Moreover, the cellular expression of p62 and LC3 proteins, which are autophagosome/autolysosomes-associated and consumed by autophagic digestion^3,4^, was increased in SphK2-deficient macrophages. The present study showed that phosphorylation of the phagophore formation-stimulating factor ULK1 and the autophagosomal content-recruiting factor p62 was mildly reduced, suggesting slightly enhanced phagophore formation and attenuated recruitment of autophagosomal contents, respectively. However, the findings collectively suggested that SphK2-deficient macrophages primarily suffered from diminished autophagic breakdown of LDs.

OxLDL is endocytosed and after oxLDL-loaded endosomes fuse with lysosomes, OxLDL is degraded by lysosomal enzymes. Released free cholesterol is transported into the endoplasmic reticulum and processed into LDs, which is released into the cytoplasm. The present study showed that lipid processing in the autophagic pathway is impaired in SphK2^*−/−*^ macrophages very likely due to lysosomal dysfunctions, resulting in more accumulation of lipids. The lysosomal dysfunctions in SphK2^*−/−*^ macrophages suggest that lysosomal degradation of endocytosed oxLDL may be impaired. Several recent studies^16,19^ suggested the involvement of SphK1 in endocytic process. However, it is currently unknown whether SphK2 is involved in the regulation of endocytosis of oxLDL and its lysosomal processing.

SphK2-deficient macrophages accumulated sphingosine and ceramides within cells, which led to a disturbance in the membrane lipid composition. A previous study^22^ showed that SphK2-deficiency induced a compensatory autophagic response, which eliminated sphingolipids that accumulated in the cell membrane due to SphK2 deficiency. In our experimental condition, macrophages were exposed to further stress of increased lipid-burden due to hyperlipidemia. Despite that SphK2-deficient macrophages sought to compensate for lipid-overloading by enhancing an autophagic response through the mechanisms involving the upregulation of TFEB, the master transcription factor for expression of autophagy- and lysosome-regulating genes, enhancement of autophagic degradation was not sufficient to prevent accumulation of the LDs in SphK2-deficient macrophages. Thus, our data imply that disturbed sphingolipid metabolism due to SphK2 deficiency compromises the autophagosome-lysosomal functions at least in macrophages, leading to excess accumulation of lipids in macrophages. A recent study^32^ showed that SphK2 but not SphK1 is involved in preconditioning-induced protection from brain injury through an autophagy-dependent mechanism. Therefore, it is an intriguing possibility that SphK2 may modulate various pathological conditions through stimulating autophagy.

Sphingosine, which has the amino group in the molecule, is recognized as a lysosomotropic substance^18,19^. In the lysosomes where the bulk of sphingosine is generated by sphingolipid degradation^9^, SphK2 deficiency results in sphingosine accumulation, which probably increases lysosomal membrane permeability like the cases of increased production of reactive oxygen species and stimulation with exogenous inflammatory cytokines, leading to lysosomal dysfunctions^33,34^. Once the lysosomal membrane is disrupted, the lysosomes release cathepsins, leading to further damages of the lysosomes and other organelles through a vicious cycle^35^. Sphingosine accumulation in the lysosomes was also shown to affect the luminal Ca^2+^ concentration and Ca^2+^ release in the lysosomes, which may change the activation of TFEB^18,34,36^. In addition, defective autophagy was reported to lead to activation of inflammasomes, which could enhance the production of inflammatory cytokines^28^. Consistent with this notion, we observed enhancement of lipopolysaccharide-induced IL-1β release in *SphK2*^*−/−*^ mice, which may contribute to aggravation of atherosclerosis.

Previous studies showed that pharmacological interventions to target the biosynthesis and actions of sphingolipids and genetic manipulations exerted beneficial effects on atherosclerosis. Myriocin is a potent inhibitor of serine palmitoyltransferase, which is the rate-limiting enzyme of the de novo sphingolipid biosynthetic pathway. Administration of myriocin decreased the concentrations of blood sphingolipids including ceramides and sphingomyelin and substantially inhibited atherosclerosis^7,37^. FTY720 (fingolimod), which was originally derived from myriocin, is a prodrug and its phosphorylated form acts as an agonist for all S1P receptor subtypes but S1PR2. FTY720 was found to reduce circulating lymphocytes via S1PR1, exerting immunosuppressive effects, and is now employed for treatment of multiple sclerosis^38^. In a murine model of atherosclerosis, chronic administration of FTY720 and S1PR1-selective agonists reduced atherosclerotic lesions^12,13,39,40^, and a more recent study^41^ showed that endothelial-specific genetic deletion of *S1pr1* exacerbated atherosclerosis, indicating that endothelial S1PR1 limits atherosclerosis. In addition, genetic deletion of either *S1pr2* or *S1pr3* diminished atherosclerosis associated with suppression of macrophage infiltration in a murine model^10,11^, indicating that S1PR2 and S1PR3 facilitate atherosclerosis.

Very recently, low density lipoprotein receptor (LDLR)-deficient chimera mice that had been transplanted with bone marrow of *Sphk2*^*−/−*^ mice were reported to show attenuated atherosclerosis^42^. The chimera mice with *Sphk2*^*−/−*^ bone marrow exhibited a moderate increase (1.5- to 2.0-fold) in plasma S1P concentration compared with wild-type bone marrow-transplanted control mice. These authors suggested that increased endogenous S1P levels exerted anti-atherogenic effects that were mediated by favorable modulation of endothelial functions. In our study, plasma S1P concentration in *Sphk2*^*−/−*^ mice was approximately 4.0-fold higher compared with wild-type mice. It could be possible that the higher S1P levels might exert enhanced anti-atherogenic effects via S1PR1. The higher S1P levels might also exert enhanced pro-atherogenic effects via S1PR2 and S1PR3. These anti-atherogenic and pro-atherogenic effects might cancel each other out. Further, since overexpression of SphK1 in *Sphk2*^*−/−*^ mice rescued both the macrophage lysosomal dysfunctions and aggravation of atherosclerosis in the present study, it is unlikely that slightly enhanced activation of S1PR2 and S1PR3 by the elevated plasma S1P could account for aggravated atherosclerosis in *Sphk2*^*−/−*^ mice. It is also possible that the different atherogenic mouse models employed in these two studies (LDLR-KO vs. ApoE-KO) might affect the discrepancies of the experimental results. Further studies including analysis of macrophage-specific *Sphk2* KO mice are required for fully understanding a role of SphK2 in atherosclerosis.

In the present study, the effects of genetic deletion of *Sphk2* and *Sphk1* on atherosclerosis were clearly different. Previous studies on SphK-deficient mice showed that the two SphK isoforms possess partially redundant functions in organismal development^15^. The SphK isoforms also play differential roles in various pathological conditions, including cancer^43,44^, inflammatory diseases^45^, and ischemic diseases^46^. SphK1 and SphK2 are expressed ubiquitously in a variety of tissues, but with different tissue abundance^47^. The abundance of the two SphKs at cellular levels is also variable^48^. In macrophages, the SphK2 mRNA level is higher than the SphK1 mRNA level (Supplemental Fig. S5c), which was consistent with the results in the previous report^22^. Although the subcellular localization of the two SphK isoforms is reportedly different, we found that both SphK1 and SphK2 are best colocalized with LAMP2-positive puncta, among the various organelles examined in macrophages (Fig. 2e), which is consistent with the fact that the bulk of sphingosine is generated by sphingolipid degradation in lysosomes^49^. Furthermore, *Sphk2* deletion resulted in the accumulation of sphingosine and ceramides in macrophages (Supplemental Fig. S5a), indicating that endogenous SphK1 fails to compensate for SphK2 deficiency in macrophages. However, transgenic overexpression of SphK1 corrected the phenotypes, including cellular accumulation of sphingosine and ceramides, which were caused by SphK2 deficiency (Fig. 5), suggesting at least partial functional redundancy of SphK1 and SphK2 in the LD catabolism. Based on these results, we reasoned that the predominant expression of SphK2 leads to its distinct role in autophagy-mediated LD catabolism in macrophages, although partial qualitative non-redundancy between the actions of SphK1 and SphK2 may exist.

In the present investigation, we studied male mice to avoid the effects of estrogen. Previous reports^50–52^ showed variable effects of sex difference on atherosclerosis in the studies that explored roles and effects of various endogenous molecules and exogenous substances on atherosclerosis. Further studies using both male and female mice are required to reveal the possible effects of sex difference on the role of SphK2 in atherosclerosis.

In conclusion, we demonstrated that macrophage SphK2 plays a critical role in autophagy-lysosomal-mediated LD catabolism to protect from atherosclerotic lesion formation. Rescuing dysregulated sphingolipid metabolism and organelle dysfunctions in macrophages may be a novel therapeutic target for atherosclerosis and other autophagy-involving pathological conditions.

## Material and Methods

### Mice

*Sphk1*^−/−^ mice and *Sphk2*^*−/−*^ mice^15^ were backcrossed into the C57BL/6J background more than 10 times. *Sphk2*^*−/−*^ mice carried LacZ-Neo cassette containing an internal ribosomal entry sequence at exon 4 of the *Sphk2* gene locus and expressed *Sphk2-LacZ* hybrid transcript, which was driven by endogenous *Sphk2* promoter elements^15^. *Sphk1-Tg* mice with the C57BL/6J background were previously described^53^. *Apoe*^*−/−*^ mice (B6.129P2-Apoetm1Unc/J, Stock Number:002052) were obtained from The Jackson Laboratory (Bar Harbor, ME). All mice were maintained in a temperature-controlled conventional facility (24 °C) under a 12-h light/12-h dark cycle with ad libitum. Experiments were performed with male knockout mice and appropriate male wild-type littermate control. For atherosclerotic lesion analysis, mice were fed a western diet (1.25% cholesterol, 7.5% cocoa butter, 7.5% casein (%fat/kcal 36%); Oriental Yeast Co., Ltd (Tokyo, Japan)) and analyzed for atherosclerotic lesion size after 12-weeks of WD feeding. For BM transplantation for generating chimeric mice, the recipient *SphK2*^+/+^ mice were irradiated with 2 doses of 4.0 Gy, 3–4 h apart, and reconstituted with unfractionated BM cells (around 2 × 10^7^ cells/recipient) from donor mice. Engraftment efficiency was about 94–95% confirmed by flow cytometry. Four weeks later, WD was started. All experiments using mice were performed according to the Guidelines for the Care and Use of Laboratory Animals of Kanazawa University, which strictly conforms to NIH guidelines, and were approved by the Committee on Animal Experiments in Kanazawa University.

### Isolation and analyses of macrophages

Peritoneal macrophages were harvested from mice 3–5 days after intraperitoneal injection of 4% thioglycolate solution (#225640, BD Difco). After 2 h incubation with 10% FBS/DMEM on plastic dishes, nonadherent cells were removed by PBS wash and adherent cells were used for further experiments. For determinations of modified LDL uptake, macrophages were incubated with human OxLDL (50 μg/ml, #RP-049, Intracel Resources) in 10% FBS/DMEM for 4 h followed by Bodipy 493/503 staining (1:1000, #D3922) (Molecular Probes) for visualization of LDs. For cholesterol efflux assays, cells were labeled with 2 μCi/ml [1,2–3 H(N)]-cholesterol (Perkin Elmer) overnight. After washing with PBS three times, cells were equilibrated 2 h by DMEM plus 0.2% fatty acid free bovine albumin (DMEM/BSA). The cells were then incubated with fresh DMEM/BSA in the absence or presence of 2% serum or HDL (50 μg/ml) at 37 °C for 4 h. The media were collected and counted for radioactivity by liquid scintillation counter. The cells were solubilized by 0.1 N NaOH and the residual radioactivity was determined. The percent efflux was calculated as [(cpm in media)/(cpm in media + cpm in cells)] × 100. Cholesterol contents were measured in cell extracts using the Amplex Red Cholesterol assay kit (A12216, Molecular Probes) and adjusted by cell protein contents determined with Bradford method (#500–0006) (Bio-Rad). For amino acid starvation, cells were incubated by 10% FBS/DMEM without amino acid (#048-33575) (WAKO, Osaka, Japan) after OxLDL uptake in the normal growth medium. To evaluate LDs and lysosomal functions, Bodipy 493/503, DQRed-BSA (#D12051) (Molecular Probes) and LysoTracker Red (#L5728) (Molecular Probes) were used according to the instruction manuals from the manufacturers.

### Histology and immunostaining

Aortae from the origin to the aortoiliac bifurcation were harvested. Spread aortae and cross-sections of aortic sinus and abdominal aorta were stained with ORO. The ORO-positive area over total en face are in each aorta was quantified using ImageJ (NIH) software. Immunofluorescent staining of aortic cryosections and macrophages were performed as described previously^54^ with the following antibodies: SphK1 (rabbit polyclonal, kindly donated by Dr. Yoshiko Banno)^49^, SphK2 (Proteintek #17096-1-AP), p62 (#610832, BD Biosciences), LAMP2 (#550292, BD Biosciences), F4/80 (MCA497RT, Abd Serotec), LC3A/B (#12741, Cell signaling technology (CST)), LC3 mAb (#M186-3, MBL (Nagoya, Japan)), EEA1 (#610456, BD Biosciences), Rab7 (R8779, Sigma Aldrich). Cell nuclei were counterstained with 4′6-diamidino-2-phenylindole (DAPI) (#D1306, Molecular Probes). Stained specimens were observed by confocal fluorescence microscope (LSM 510 Pascal; Carl Zeiss) and quantified by using ImageJ or ZEN 2.1 software (Carl Zeiss).

### X-gal staining

For whole mount tissue X-gal staining, mice were perfused with saline and tissues were fixed in freshly prepared 0.25% glutaraldehyde solution (2 mM MgCl2, 5 mM EDTA) at room temperature for 30 min. After brief rinse by Ca^2+^- and Mg^2+^-free PBS, tissues were washed 3 times by detergent rinse buffer (0.1 M phosphate buffer (pH 7.3), 2 mM MgCl2, 0.01% DOC, 0.02% NP-40) 30 min each. Finally, tissues were stained by 1 mM X-gal solution (5 mM potassium ferricyanide, 5 mM potassium ferrocyanide) in detergent rinse buffer overnight at 37 °C. Tissues were post–fixed by 4% PFA for 30 min and stored in PBS for photo capture. For microscopic observations of X-gal-stained tissues, tissue sections were prepared from OCT compound-embedded tissues and counterstained with Nuclear fast red.

### Western blot analysis

Aortae were lysed in RIPA buffer (50 mM Tris-HCl pH7.4, 150 mM NaCl, 5 mM EDTA, 1% Triton X-100, 0.1% SDS, 0.5% sodium deoxycholate, 2.5 mM sodium pyrophosphate, 1 mM β-glycerophosphate, 1 mM Na3VO4 including protease inhibitors (Complete Protease Inhibitor Cocktail, Roche). Cell lysates were separated on SDS-PAGE gels and then transferred to Polyvinylidene difluoride membranes. Proteins were detected by using primary antibodies: p62 (#610832, BD Biosciences), LAMP2 (Mac3/CD107b, #550292, BD Biosciences), ABCA1 (#400-164, Novus Biologicals), ABCG1 (#AB36969, Abcam), TFEB (#A303-673A, Bethyl),GAPDH (#016-25523, WAKO) and LC3A/B (#12741), IL-1β (#12426), Beclin-1(#3495), Keap1 (#8047), p-4EBP1 (Thr^37/46^, #2855), p-S6K (Thr^389^) (#9234), p-mTOR (Ser^2481^) (#2974), total mTOR (#2983), p-ULK1 (Ser^757^) (#14202), total ULK1 (#8054), p-p62 (Ser^403^) (#39786) from CST. Proteins were visualized by chromogenic reaction using alkaline phosphatase-conjugated secondary antibody and NBT/BCIP substrate.

### Quantitative real-time PCR

Total RNA was extracted by RNeasy Liquid Tissue Mini Kit (Invitrogen) and reverse-transcribed with a RT2 First Strand Kit or QuantiTect Reverse Transcription Kit (Qiagen). Sequences of target primers are listed in Supplemental Table S1. Real-time quantitative PCR was performed using the ABI PRISM 7300 sequence detection system (Applied Biosystems) with RT2 SYBR GREEN qPCR MasterMix (QIAGEN) or FastStart Universal SYBR Green Master ROX (Roche)^55^. The relative quantity of interest gene mRNA normalized by GAPDH mRNA (2^-ΔΔCt^) was analyzed.

### Foam cell analysis by FACS

Blood was drawn from WD-fed mice in the presence of anti-coagulant (EDTA-2K). After Fc receptor blocking (#553141, BD), cells were stained with fluorescently labelled antibodies F4/80-Alexa488 (#MF48020, 1:20, invitrogen), CD11b-PE (#561689, 1:20, BD) and CD45-PerCP (#561047, 1:20, BD Biosciences) followed by FACS Lysing Solution (#349202, BD Biosciences), according to the instruction manual. Circulating foam cell cluster in high SSC subsets of F4/80^+^CD11b^+^CD45^+^ were measured by Attune Acoustic Focusing Cytometer (Applied biosystems) with modifications as reported previously^56^.

### Biochemical analysis

Whole blood drawn with EDTA-2K was centrifuged by 5 min at 10,000 rpm at 4 °C. Resultant plasma was removed carefully, aliquoted and stored at −80 °C until analysis. Total cholesterol and triglycerides were determined with AutoAnalyzer (SRL Inc.). For lipoprotein fraction analysis, HPLC system with 2 tandem gel permeation columns was used to evaluate the size distribution of plasma lipoprotein particles (Skylight Biotech Inc). Plasma S1P concentration and sphingolipid profile of mouse macrophages were determined by HPLC and LC-MS/MS, respectively as described before^57,58^.

### Enzyme-linked immunosorbent assay

The murine plasma IL-1β was measured by DuoSet ELISA kit (DY401-05, R&D systems) according to the manufacturer’s instructions. 1-Step™ Ultra TMB-ELISA (Thermo scientific, cat# 34028) was used for final horseradish peroxidase reaction and terminated by 1 M sulfuric acid.

### Statistical analysis

All data are represented as the means ± s.e.m. At least, 2 independent experiments were performed unless otherwise indicated. Statistical significance was determined using one-way ANOVA with Turkey’s post hoc test for pairwise comparison or the unpaired two-tailed Student’s t-test with Prism version 6 software (GraphPad Software Inc.). P < 0.05 was considered significant different and Welch’s correction was applied when variances are significantly different in the Student’s t-test.

## Supplementary information

Supplementary information

## Data Availability

The data that support the findings of this study are available from the corresponding authors on reasonable request.
