# Supplementary material for: Sphingosine kinase-2 prevents macrophage cholesterol accumulation and atherosclerosis by stimulating autophagic lipid degradation
Source: Sci Rep. 2019 Dec 4;9:18329. doi: 10.1038/s41598-019-54877-6 (PMC6892873; doi:10.1038/s41598-019-54877-6)
Supplement: Supplementary file 1 — Supplementary information [file 41598_2019_54877_MOESM1_ESM.pdf]

Supplementary information

Sphingosine kinase-2 prevents macrophage cholesterol accumulation and atherosclerosis by stimulating autophagic lipid degradation

Kazuhiro Ishimaru, Kazuaki Yoshioka, Kuniyuki Kano, Makoto Kurano, Daisuke Saigusa, Junken Aoki, Yutaka Yatomi, Noriko Takuwa, Yasuo Okamoto, Richard L. Proia, Yoh Takuwa

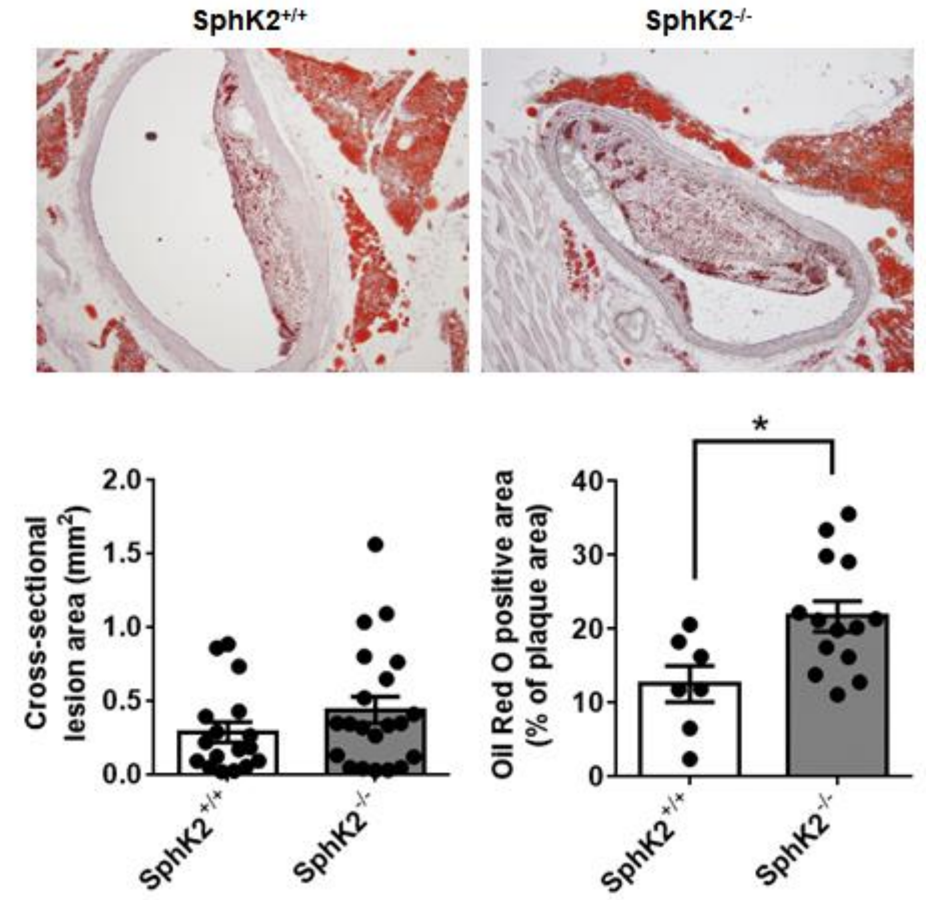

Supplemental Figure S1. Atherosclerotic lesion size and lipid-deposited areas in the cross-sections of the abdominal aorta of *SphK2*$^{+/+}$ and *SphK2*$^{-/-}$ mice. Male mice were fed a WD for 12 weeks, and the harvested aortae were stained with ORO. Seven to 14 atherosclerotic lesions from 3 mice per group were analyzed. Results are represented as the means ± s.e.m. Statistical significance was determined using the unpaired two-tailed Student's t-test for independent samples. *P<0.05.

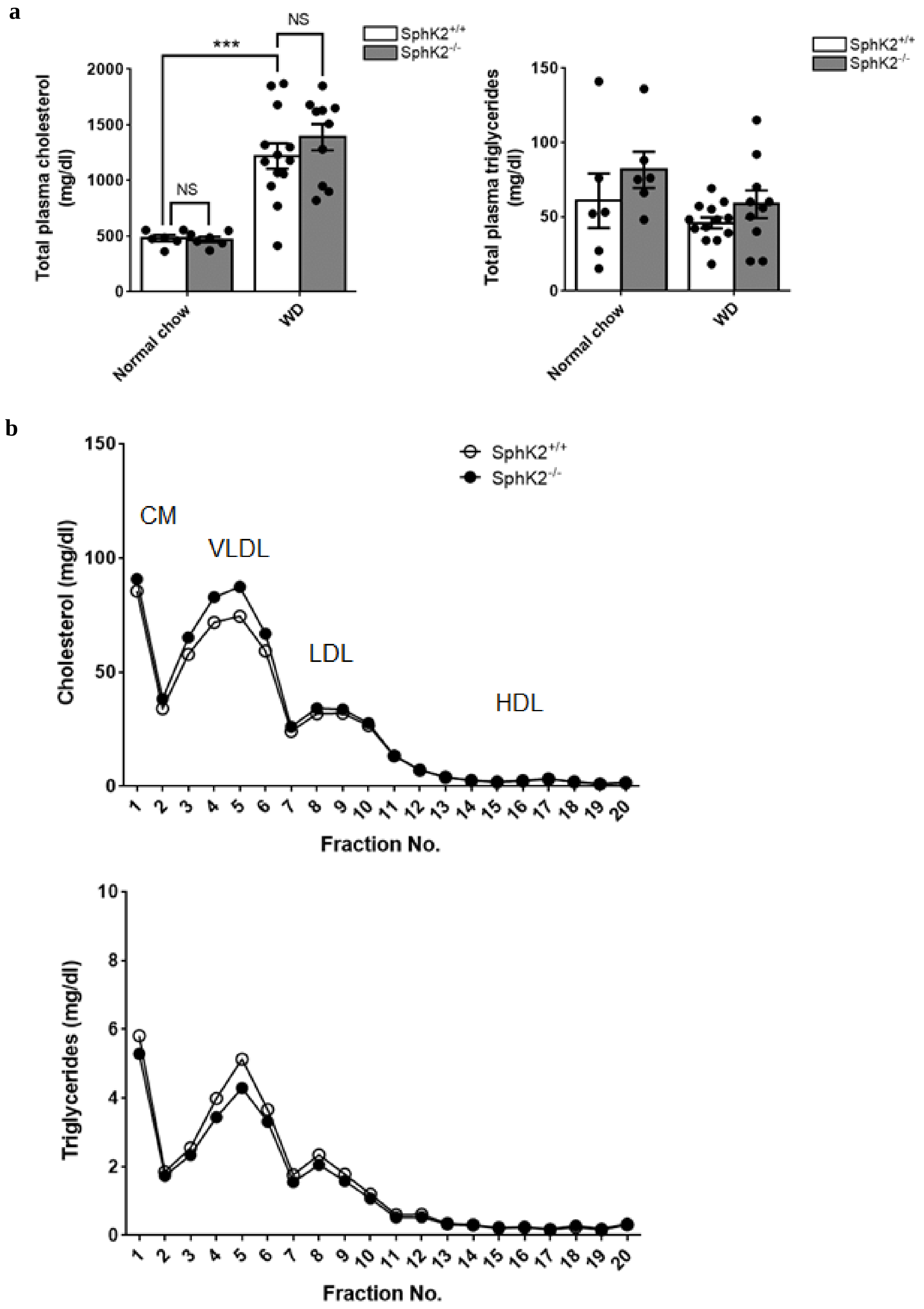

Supplemental Figure S2. Plasma lipid concentrations and lipoprotein profile in *SphK2*$^{+/+}$ and *SphK2*$^{-/-}$ mice. **a**) Total plasma cholesterol and triglyceride concentrations in male mice fed normal chow or a WD for 12 weeks (n=3-6 mice per group). **b**) Lipoprotein fractions in the plasma of male mice fed a WD for 12 weeks (n=5 mice per group). Statistical significance was determined using one-way ANOVA with Turkey's post hoc test for pairwise comparison of total cholesterol in **a**, and two-way ANOVA with Sidak's multiple comparison test in **b**. ***P<0.001. NS, not significant. Results are represented as the means ± s.e.m.

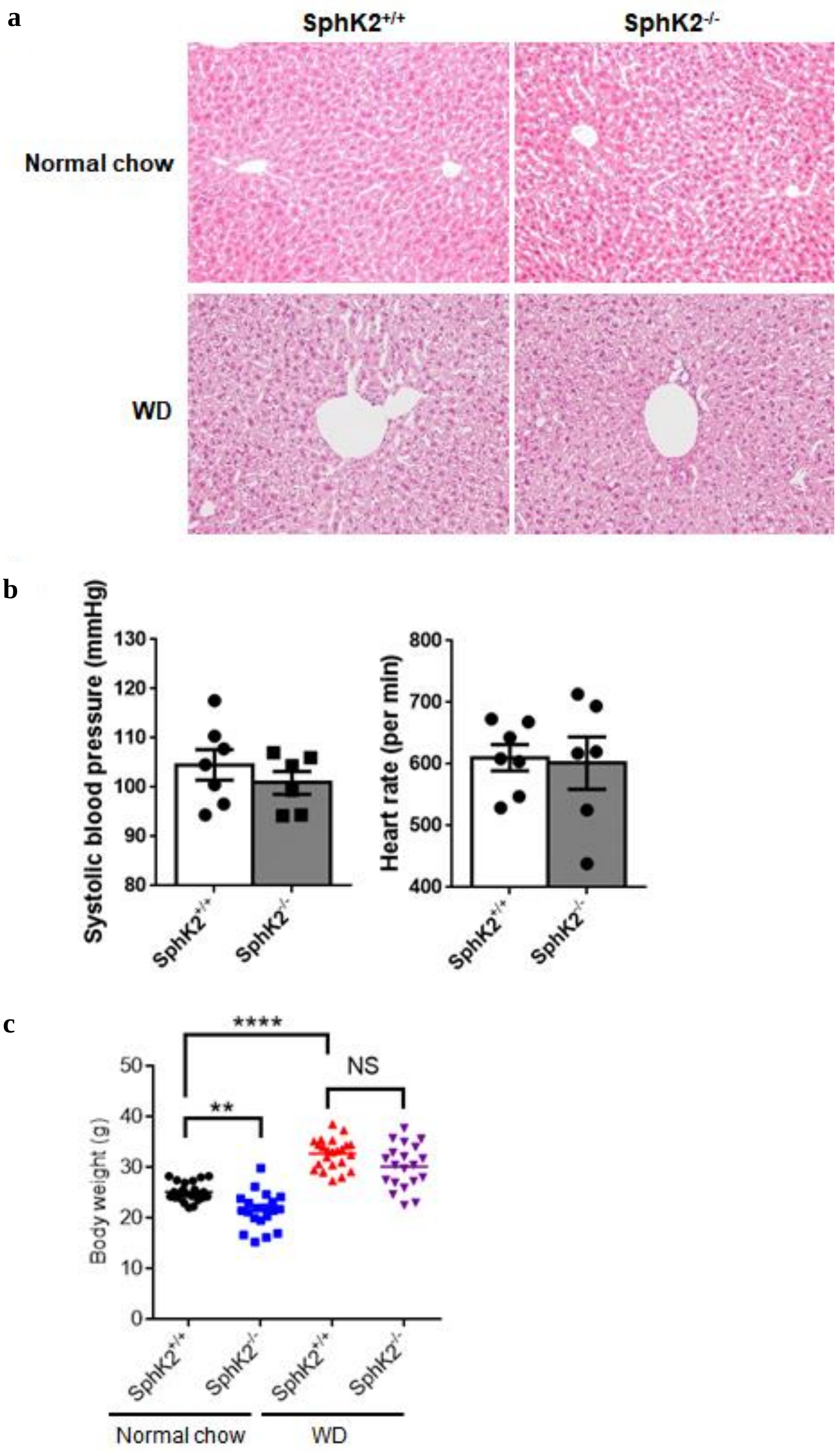

Supplemental Figure S3. Liver histology, body weight, blood pressure and heart rate of *SphK2*$^{+/+}$ and *SphK2*$^{-/-}$ mice. a) Liver histology (HE staining) of SphK2$^{+/+}$ and SphK2$^{-/-}$ male mice that were fed either normal chow or a WD (n=6-9 per group). **b)** Blood pressure and heart rate in SphK2$^{+/+}$ and SphK2$^{-/-}$ male mice after 12 weeks of a WD (n=6-7 per group). **c)** Body weights of SphK2$^{+/+}$ and SphK2$^{-/-}$ male mice at 8 and 20 weeks. Mice at 20-weeks were fed a WD for 12 weeks. Statistical significance was determined using the unpaired two-tailed Student's t-test for independent samples in **b**, and one-way ANOVA with Turkey's post hoc test for pairwise comparison in **c**. ** P<0.01 and **** P<0.0001. NS, not significant. Results are represented as the means ± s.e.m.

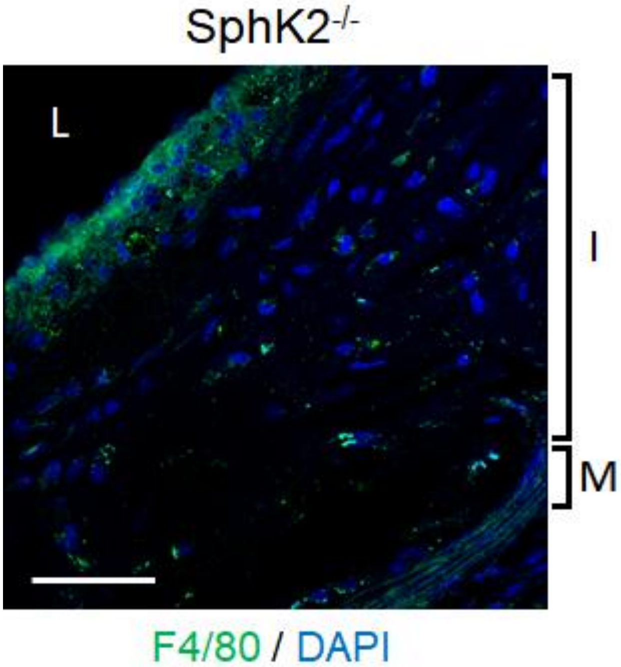

Supplemental Figure S4. F4/80 expression in the aortic root plaque. A section of the aortic root from *SphK2-/-* mouse after 12weeks of WD feeding was subjected to immunofluorescent staining using anti-F4/80 antibody. L, lumen. I, intima. M, media. Scale bars, 50 µm.

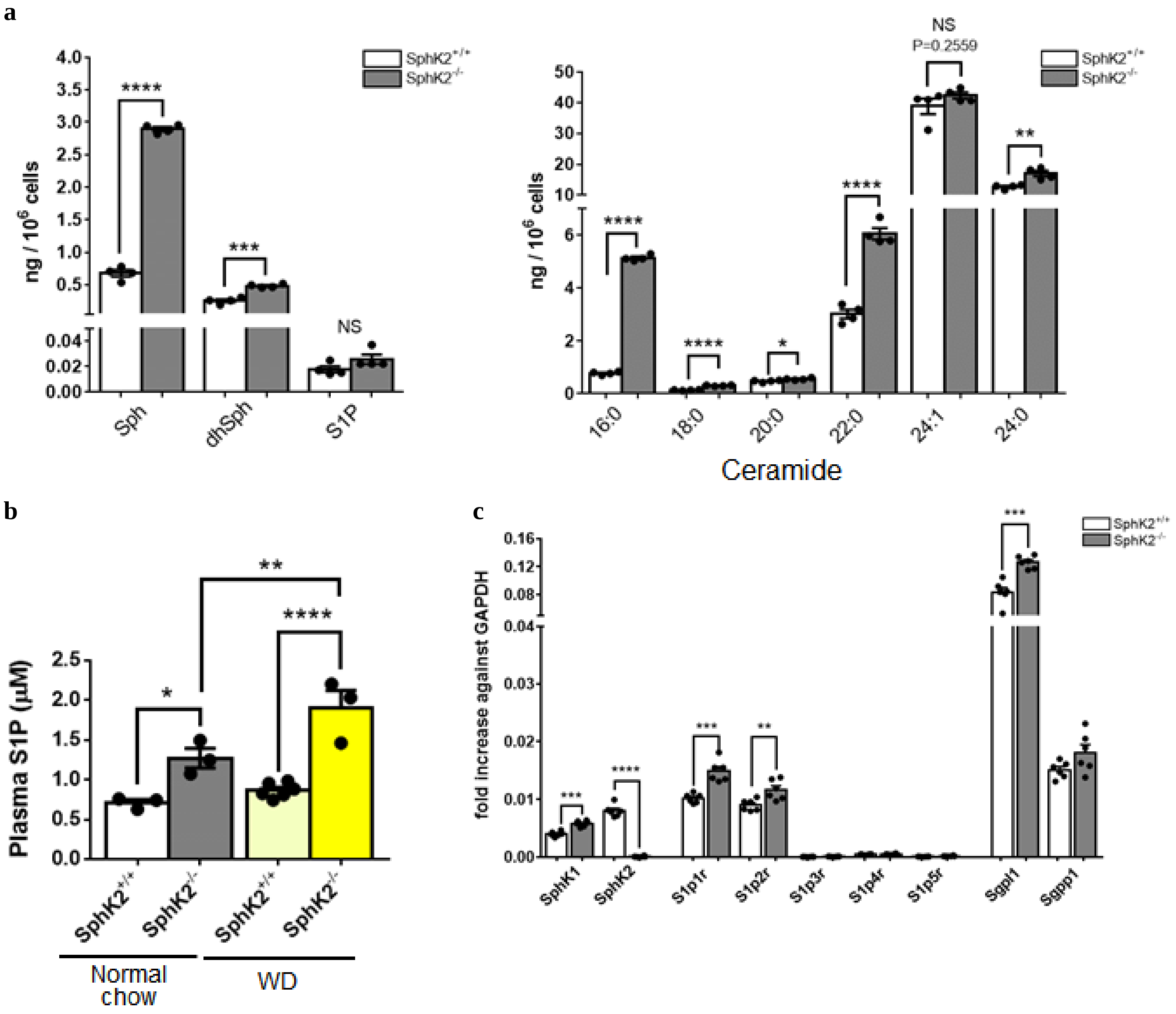

Supplemental Figure S5. Levels of sphingosine and ceramide in macrophages and plasma S1P concentrations are higher in *SphK2*$^{-/-}$ mice. a) Sphingolipid profile of peritoneal macrophages freshly harvested from WD-fed SphK2$^{+/+}$ and SphK2$^{-/-}$ male mice (n=4 per group). Sph: sphingosine, dhSph: dihydrosphingosine. **b**) Plasma S1P concentrations in male mice with or without WD feeding. **c**) Quantitative RT-PCR analysis of genes of two sphingosine kinase isoforms, five sphinogsine-1-phosphate receptors (S1pr1-5), Sgpl1 and Sgpp1 in macrophages freshly harvested from WD-fed SphK2$^{+/+}$ and SphK2$^{-/-}$ male mice (n=6 per group). Statistical significance was determined using the unpaired two-tailed Student's t-test for independent samples in **a**, and one-way ANOVA with Turkey's post hoc test for pairwise comparison in **b** and **c**. * P<0.05, ** P<0.01, *** P<0.001 and **** P<0.0001. Results are represented as the means ± s.e.m.

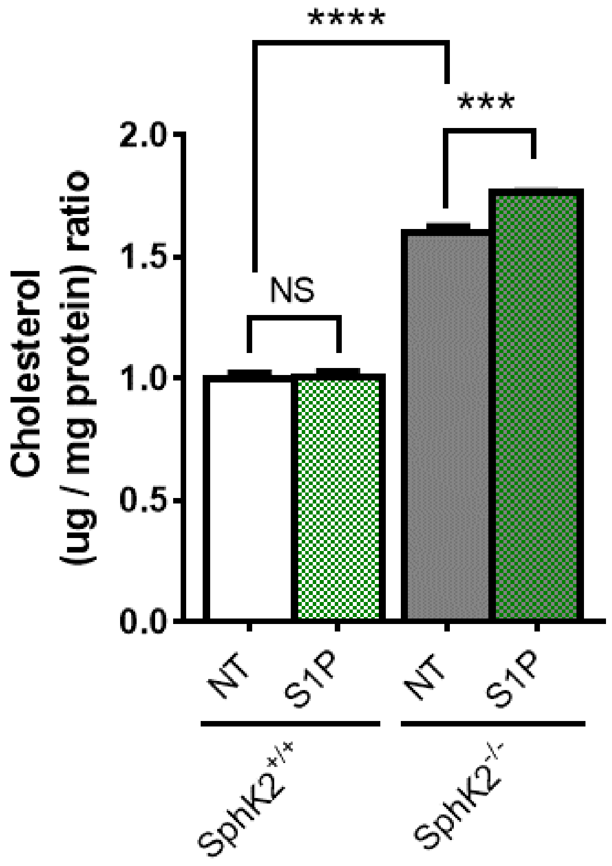

Supplemental Figure S6. The effects of treatment with exogenous S1P on cellular cholesterol contents in macrophages from *SphK2*$^{+/+}$ and *SphK2*$^{-/-}$ mice.

Cells were treated with S1P (1 μM) for 1 h, followed by the determination of cellular cholesterol. n=3 per group. Statistical significance was determined using the unpaired two-tailed Student’s t-test for independent samples. *** P<0.001 and **** P<0.0001. Results are represented as the means ± s.e.m.

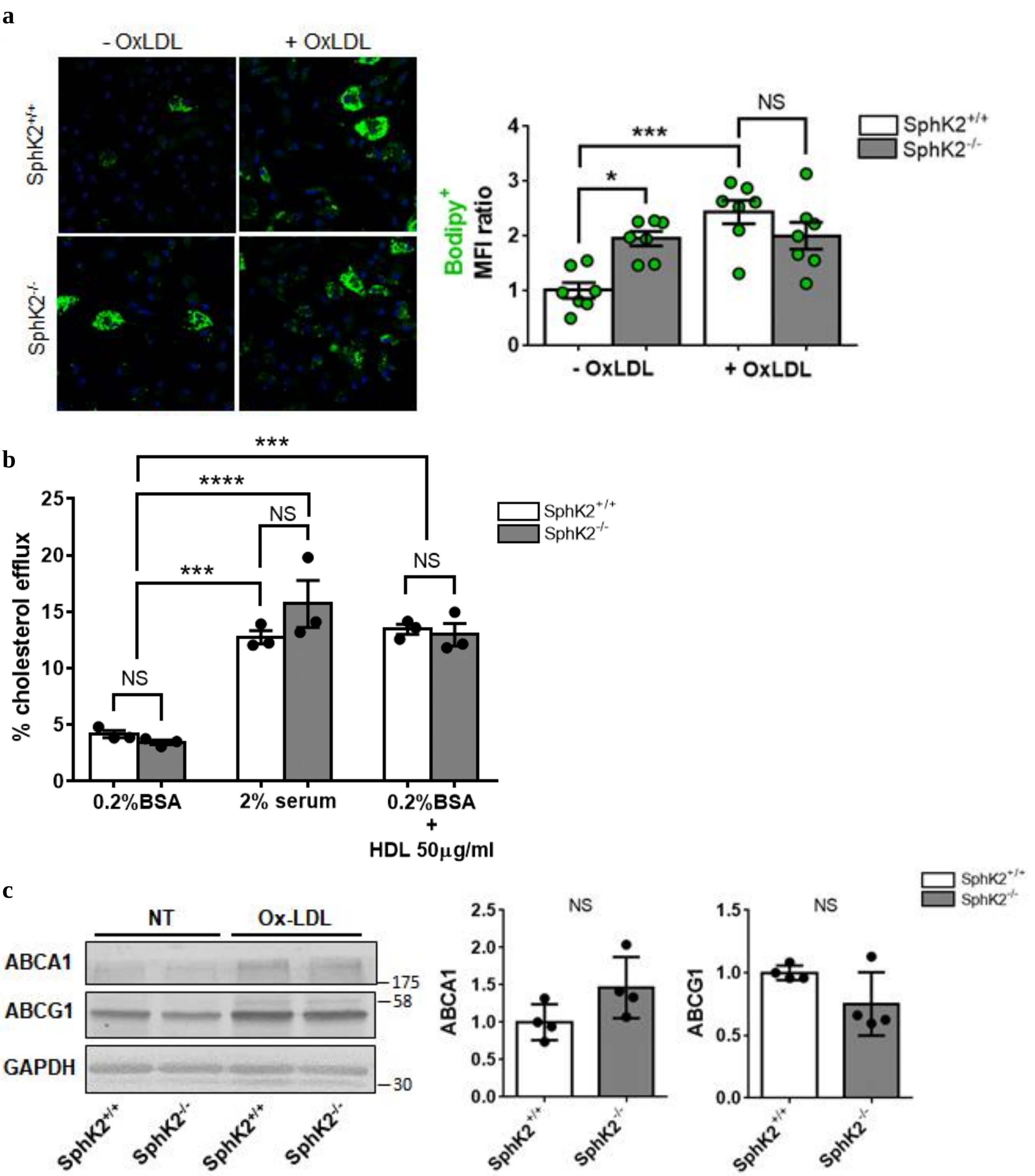

Supplemental Figure S7. OxLDL uptake and cholesterol efflux in macrophages from *SphK2*$^{+/+}$ and *SphK2*$^{-/-}$ mice. a) Uptake of OxLDL by macrophages freshly harvested from *SphK2*$^{+/+}$ and *SphK2*$^{-/-}$ male mice. Macrophages were incubated with OxLDL for 4 h, followed by Bodipy staining. Representative images (left). DAPI for nuclear staining. Scale bars, 20μm. MFI of Bodipy signals (right). (n=7 per group). **b**) Cholesterol efflux from macrophages freshly harvested from *SphK2*$^{+/+}$ and *SphK2*$^{-/-}$ male mice (n=3 per group). **c**) Immunoblot analysis of expression of the cholesterol efflux regulatory proteins ABCA1 and ABCG1 in *SphK2*$^{+/+}$ and *SphK2*$^{-/-}$ macrophages loaded with OxLDL or not (NT) for 24 h (n=3 per group). Statistical significance was determined using one-way ANOVA with Turkey's post hoc test for pairwise comparison in **a** and **b**, and the unpaired two-tailed Student's t-test was used for independent samples in **c**. * P<0.05 and *** P<0.001. NS, not significant. Results are represented as the means ± s.e.m.

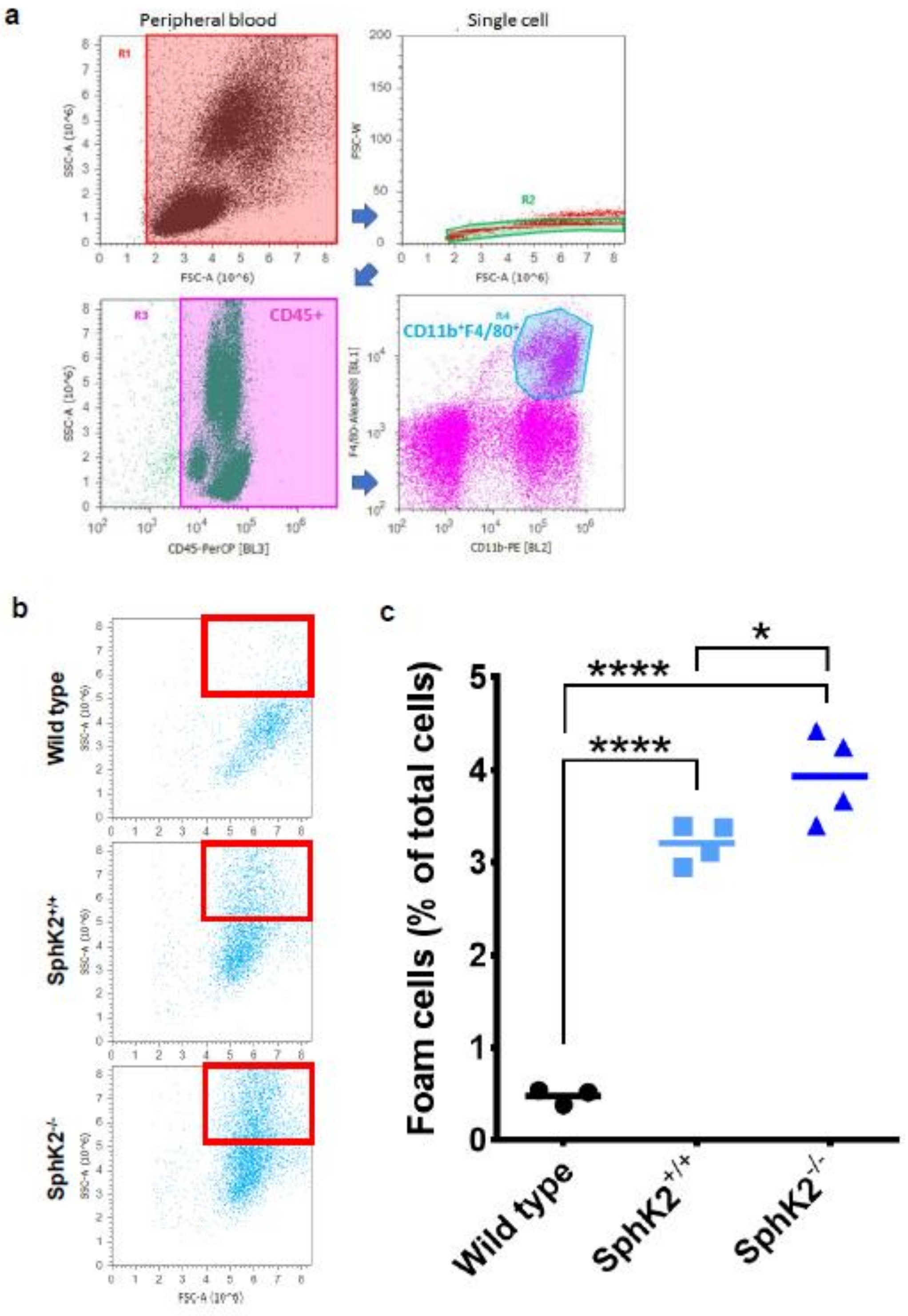

Supplemental Figure S8. Increased circulating foamy monocytes in *SphK2*$^{-/-}$ mice. a) Isolation of CD45^{+}CD11b^{+}F4/80^{+} cells (monocyte) from peripheral blood. **b**) Analysis of Side Scatter (SSC) and Forward Scatter (FSC) in CD45^{+}CD11b^{+}F4/80^{+} cells. **c**) The quantified data of high SSC subsets of CD45^{+}CD11b^{+}F4/80^{+} cells (foamy monocytes), which are expressed as the percentage of total peripheral blood nuclear cells (n=3-4 per group). **a-c**) Leukocytes of the peripheral blood from wild-type, Apoe$^{-/-}$; SphK2$^{+/+}$ and Apoe$^{-/-}$; SphK2$^{-/-}$ male mice that had been fed a WD for 12 weeks were sorted using FACS as indicated. Statistical significance was determined using one-way ANOVA with Turkey's post hoc test for pairwise comparison. * P<0.05 and **** P<0.0001.

a

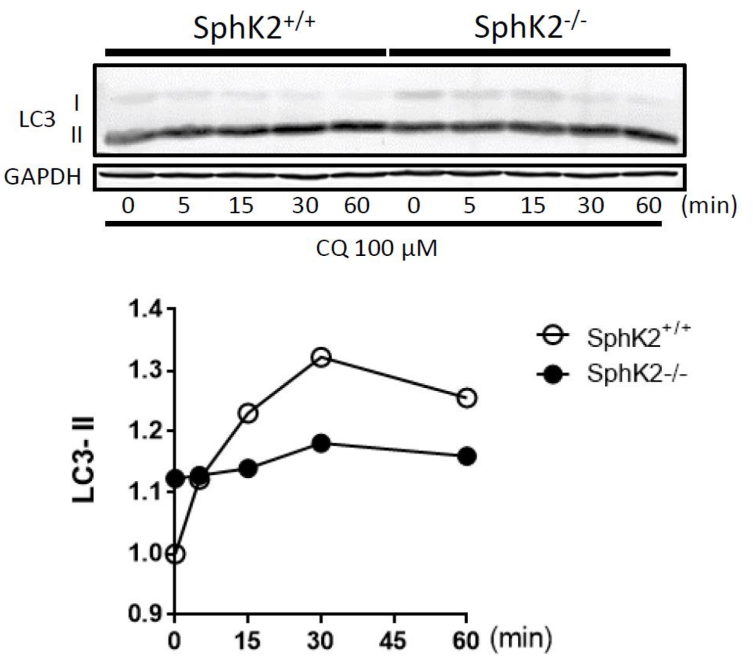
SphK2+/+
SphK2-/-
LC3
I
II
GAPDH
0 5 15 30 60 0 5 15 30 60 (min)
CQ 100 μM
LC3- II
1.4
1.3
1.2
1.1
1.0
0.9
0 15 30 45 60 (min)
SphK2+/+
SphK2-/-

b

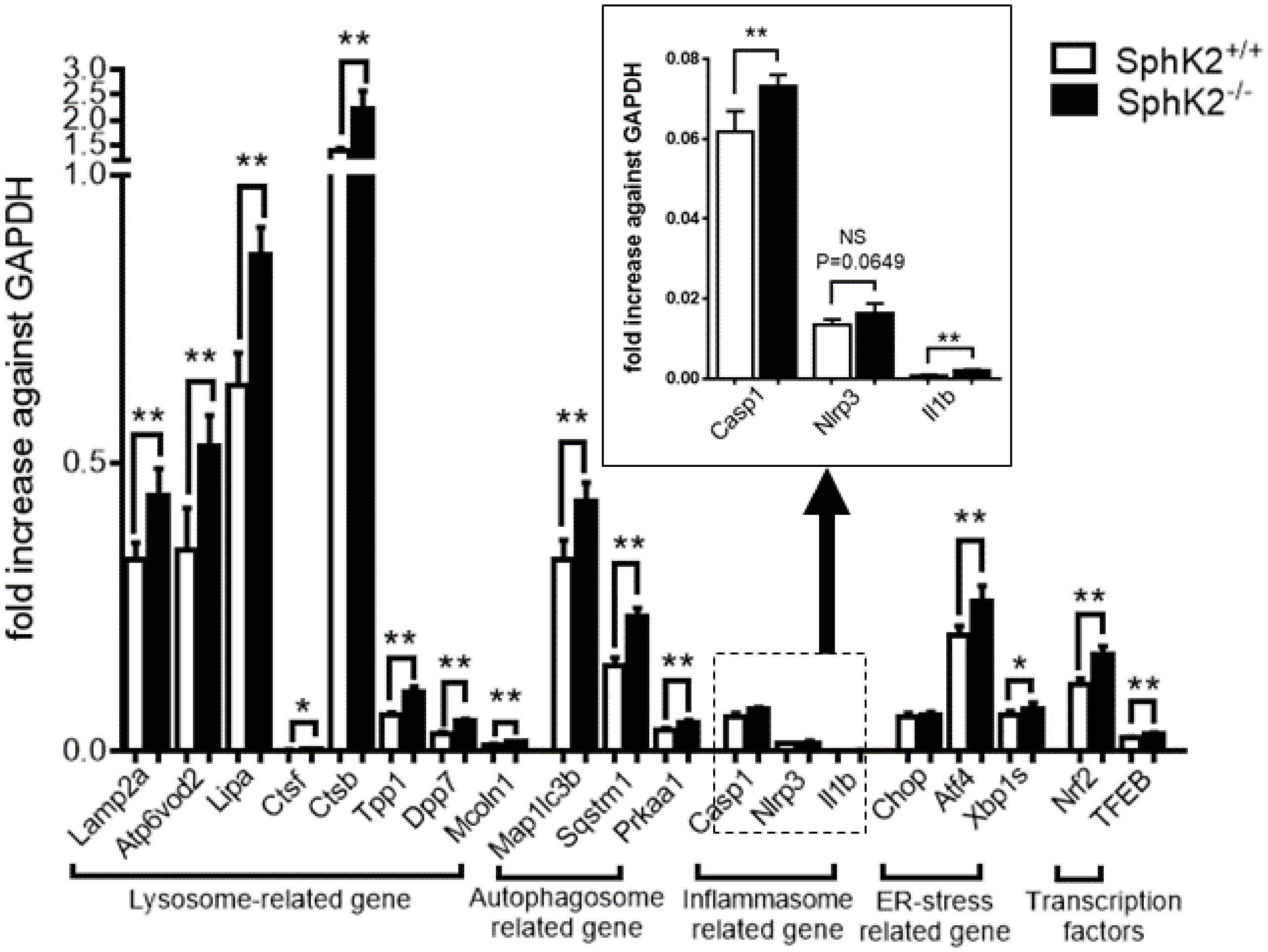
fold increase against GAPDH
3.0
2.5
2.0
1.5
1.0
0.5
0.0
SphK2+/+
SphK2-/-
**
*
NS
P=0.0649
0.08
0.06
0.04
0.02
0.00
Casp1
Nlrp3
Il1b
Lamp2a
Atp6vod2
Lipa
Ctsf
Ctsb
Tpp1
Dpp7
Mcoln1
Map1lc3b
Sqstm1
Prkaa1
Chop
Atf4
Xbp1s
Nrf2
TFEB
Lysosome-related gene
Autophagosome related gene
Inflammasome related gene
ER-stress related gene
Transcription factors

c

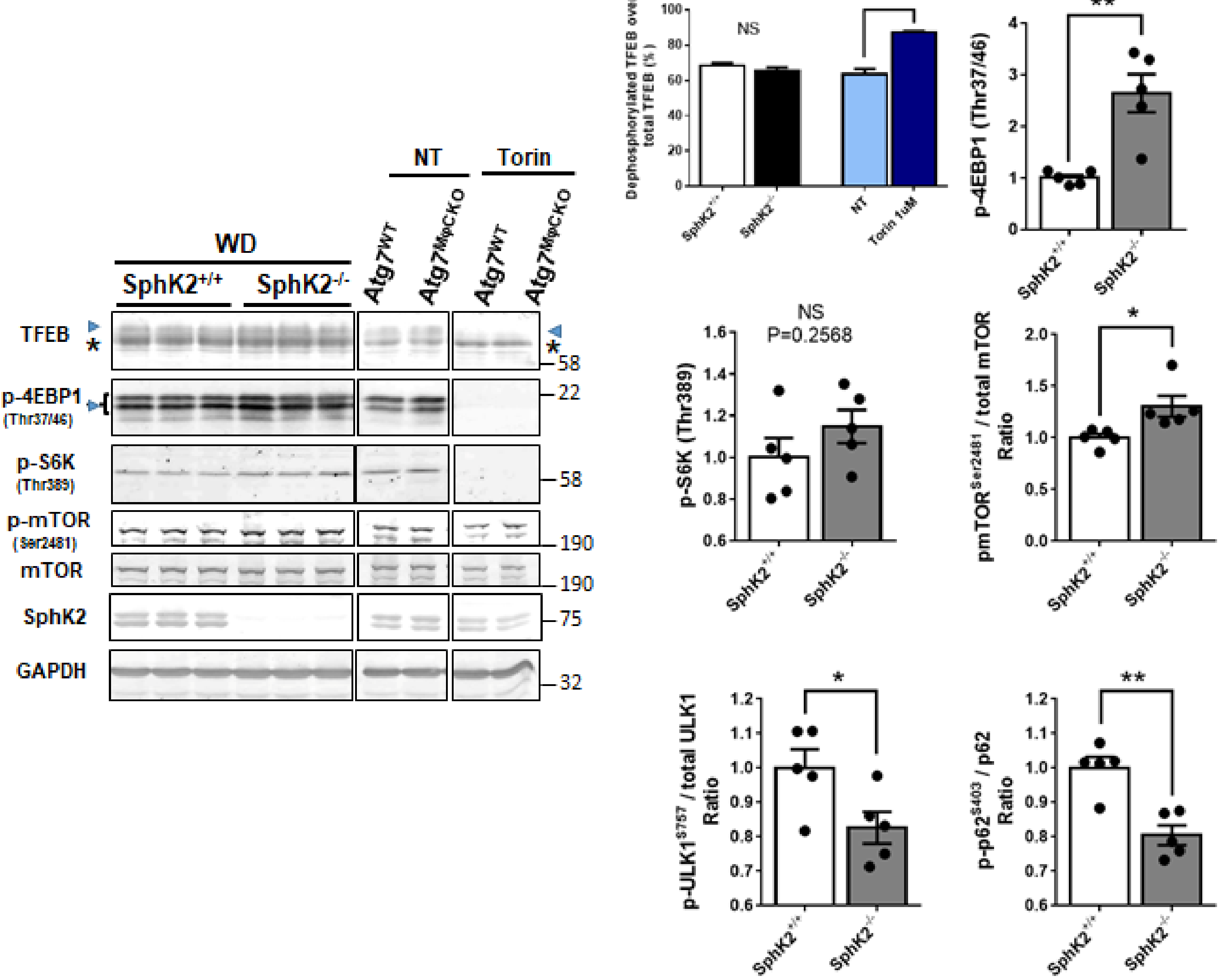
WD
SphK2+/+
SphK2-/-
NT
Torin
Atg7WT
Atg7MφCKO
TFEB
p-4EBP1
(Thr37/46)
p-S6K
(Thr389)
p-mTOR
(Ser2481)
mTOR
SphK2
GAPDH
58
22
58
190
190
75
32
Dephosphorylated TFEB over total TFEB (%)
NS
****
SphK2+/+
SphK2-/-
NT
Torin 1uM
p-4EBP1 (Thr37/46)
**
p-S6K (Thr389)
NS
P=0.2568
pmTORSer2481 / total mTOR
Ratio
*
p-ULK1S757 / total ULK1
Ratio
*
p-p62S403 / p62
Ratio
**

d

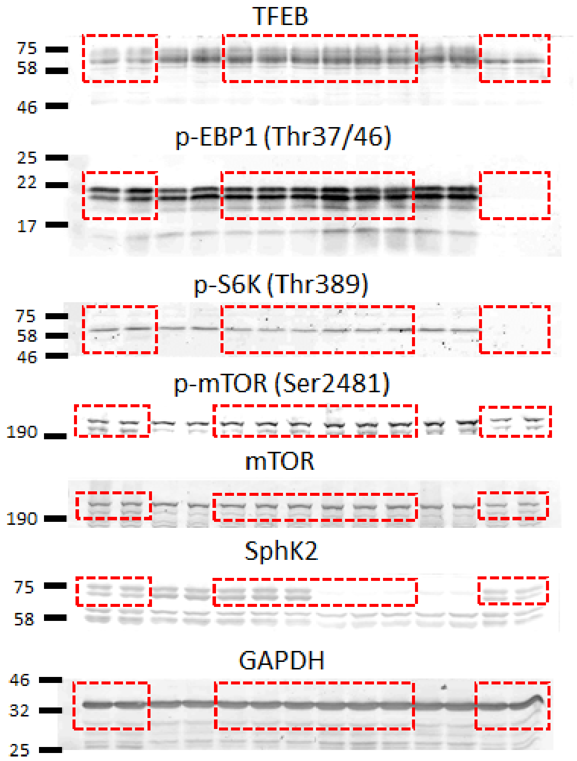
TFEB
p-EBP1 (Thr37/46)
p-S6K (Thr389)
p-mTOR (Ser2481)
mTOR
SphK2
GAPDH

e

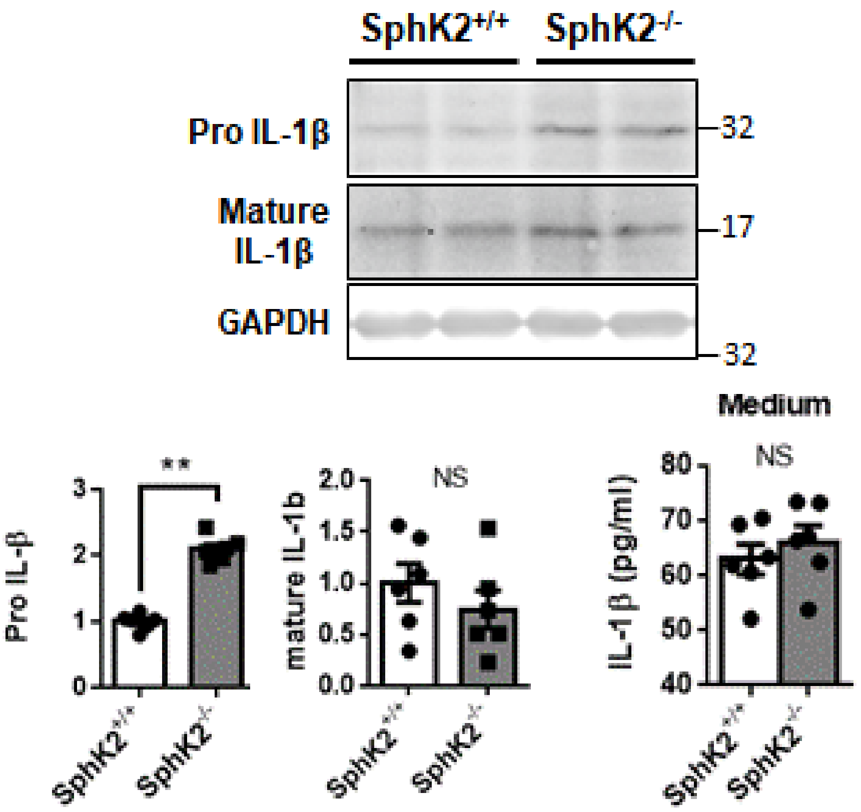

SphK2+/+
SphK2-/-
Pro IL-1β
Mature IL-1β
GAPDH
32
17
32
Medium
Pro IL-β
mature IL-1b
IL-1β (pg/ml)
**
NS
NS

f

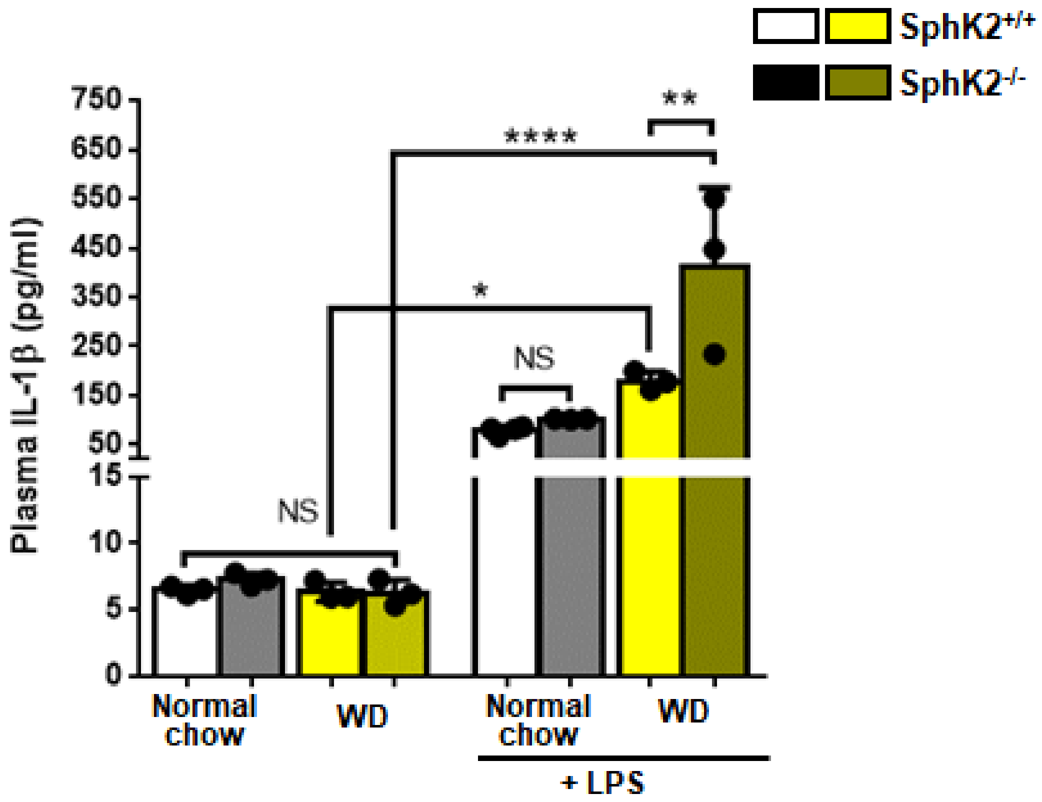

SphK2+/+
SphK2-/-
Plasma IL-1β (pg/ml)
**
****
*
NS
NS
Normal chow
WD
Normal chow
WD
+ LPS

Supplemental Figure S9. Increased expression of lysosome- and autophagy-related genes and inflammatory genes in *SphK2*$^{-/-}$ macrophages. **a**) Autophagy flux analyses by chloroquine (CQ) in macrophages from *SphK2*$^{+/+}$ and *SphK2*$^{-/-}$ mice. Cells were treated with CQ for varied time periods. **b**) Quantitative RT-PCR analysis of the lysosome-, autophagosome-, inflammasome-related genes (inset) and ER stress-related genes, and transcriptional factor genes in macrophages from *SphK2*$^{+/+}$ and *SphK2*$^{-/-}$ male mice (n=6 per group). **c**) Immunoblot analyses. Macrophages were harvested from *SphK2*$^{+/+}$ and *SphK2*$^{-/-}$ male mice, macrophage-specific conditional Atg7-deleted (Atg7$^{M\varphi CKO}$) male mice and Atg7$^{fl/fl}$ male mice. Macrophages were treated with Torin (1μM) for 1hr or non-treated (NT). The representative blot (left) and quantified data (right). The indicated grouped blots were cropped from the different blots, as shown in **d**. The arrowheads and asterisk denote phosphorylated and non-phosphorylated forms of TFEB, respectively. **d**) The full length blots used for preparing the cropped blots in **c**. The portions in the red boxes were cropped and combined in **c**. **e**) Immunoblot analyses of cellular pro IL-1β and mature IL-1β proteins, and mature IL-1β protein concentrations in the culture media by ELISA (n=6 per group). The levels of these proteins were normalized to those of GAPDH. **f**) LPS-induced increases in plasma IL-1β (n=3-4 per group). Male mice were injected intraperitoneally with LPS (40mg/kg) and sacrificed 3 h later for determination of plasma IL-1β. In **a**)-**d**), *SphK2*$^{+/+}$ and *SphK2*$^{-/-}$ male mice were fed WD for 2 weeks. Results are represented as the means ± s.e.m.

Supplemental Table S1. List of primer sequences used for qPCR analysis in this study.

Target	Sequence
S1p1r	F: AAATGCCCCAACGGAGACT<br>R: CTGATTTGCTGCGGCTAAATTC
S1p2r	F: GCCATCGTGGTGGAGAATCTT<br>R: AGGTACATTGCTGAGTGGAACTTG
S1p3r	F: GCGCATCTACTGCCTGGTCAAGTCC<br>R: AGCCAGCATGATGAACCACTGACTC
S1p4r	F: GGCTATGCCCATTGTCCAGTA<br>R: GGCTCTGAGCTAGAGAGCATGAT
S1p5r	F: CATGGCTAACTCGCTGCTGAA<br>R: AGCTGTTGGAGGAGTCTTGGTT
SphK1	F: AGGTGGTGAATGGGCTAATG<br>R: TGCTCGTACCCAGCATAGTG
SphK2	F: ACCACTTATGAGGAGAATCG<br>R: CACCACGTGGTCCATACAGC
Sgpl1	F: GTTGGGCCGCCTTGATG<br>R: GATGATCTGTTTGGTAGCTTCAACA
Sgpp1	F: CCCATTGGTGGACCTGATTG<br>R: GATGAGCGGCGCATATTTG
Lamp2a	F: CCAAATTGGGATCCTAACCTAA<br>R: TGGTCAAGCAGTGTTTATTAATTCC
Atp6vod2	F: CAGAGCTGTACTTCAATGTGGAC<br>R: AGGTCTCACACTGCACTAGGT
Lipa	F: TGTTCGTTTTCACCATTGGGA<br>R: CGCATGATTATCTCGGTCACA
Ctsf	F: ACGCCTATGCAGCCATAAAG<br>R: CTTTTGCCATCTGTGCTGAG
Ctsb	F: TTAGCGCTCTCACTTCCACTACC<br>R: TGCTTGCTACCTTCCTCTGGTTA
Tpp1	F: AAGCCAGGCCTACATAGTCAGA<br>R: CCAAGTGCTTCCTGCAGTTTAGA
Dpp7	F: CGCCAGCAATACTGTCTGGATAC<br>R: AAATGATGTTGCTGGCTGCTTTA
Mcoln1	F: GCGCCTATGACACCATCAA<br>R: TATCCTGGACTGCTCGAT
Map1lc3b	F: CGTCCTGGACAAGACCAAGT<br>R: ATTGCTGTCCCGAATGTCTC
Sqstm1	F: ATGTGGAACATGGAGGGAAGA<br>R: GGAGTTCACCTGTAGATGGGT
Prkaa1	F: GTCAAAGCCGACCCAATGATA<br>R: CGTACACGCAAATAATAGGGGTT
Casp1	F: ACAAGGCACGGGACCTATG<br>R: TCCCAGTCAGTCCTGGAAATG
Nlrp3	F: ATTACCCGCCCGAGAAAGG<br>R: TCGCAGCAAAGATCCACACAG
Il1b	F: CAGGCAGGCAGTATCACTCA<br>R: AGGCCACAGGTATTTTGTCG
Chop	F: CTGGAAGCCTGGTATGAGGAT<br>R: CAGGGTCAAGAGTAGTGAAGGT
Atf4	F: CCTGAACAGCGAAGTGTTGG<br>R: TGGAGAACCCATGAGGTTTCAA
Xbp1s	F: AGCAGCAAGTGGTGGATTTG<br>R: GAGTTTTCTCCCGTAAAAGCTGA
Nrf2	F: TCTTGGAGTAAGTCGAGAAGTGT<br>R: GTTGAAACTGAGCGAAAAAGGC
TFEB	F: GCGAGAGCTAACAGATGCTGA<br>R: CCGGTCATTGATGTTGAACC
GAPDH	F: GACTTCAACAGCAACTCCCAC<br>R: TCCACCACCCTGTTGCTGTA
